# Transcriptomic Comparison Reveals Candidate Genes for Triterpenoid Biosynthesis in Two Closely Related *Ilex* Species

**DOI:** 10.3389/fpls.2017.00634

**Published:** 2017-04-28

**Authors:** Lingling Wen, Xiaoyun Yun, Xiasheng Zheng, Hui Xu, Ruoting Zhan, Weiwen Chen, Yaping Xu, Ye Chen, Jie Zhang

**Affiliations:** ^1^Key Laboratory of Chinese Medicinal Resource from Lingnan, Research Center of Chinese Herbal Resource Science and Engineering, Guangzhou University of Chinese MedicineGuangzhou, China; ^2^Zhongshan Zhongzhi Pharmaceutical Group, Key Laboratory for Technologies and Applications of Ultrafine Granular Powder of Herbal Medicine, State Administration of Traditional Chinese MedicineZhongshan, China

**Keywords:** *Ilex pubescens*, transcriptome, triterpenoid saponins, biosynthesis, transcriptomic comparison, gene identification, oxidosqualene cyclase, Ilex asprella

## Abstract

Native to Southern China, *Ilex pubescens* and *Ilex asprella* are frequently used in traditional Chinese medicine. Both of them produce a large variety of ursane-type triterpenoid saponins, which have been demonstrated to have different pharmacological effects. However, little is known about their biosynthesis. In this study, transcriptomic analysis of *I. pubescens* and comparison with its closely related specie *I. asprella* were carried out to identify potential genes involved in triterpenoid saponin biosynthesis. Through RNA sequencing (RNA-seq) and *de novo* transcriptome assembly of *I. pubescens*, a total of 68,688 UniGene clusters are obtained, of which 32,184 (46.86%) are successfully annotated by comparison with the sequences in major public databases (NCBI, Swiss-Prot, and KEGG). It includes 128 UniGenes related to triterpenoid backbone biosynthesis, 11 OSCs (oxidosqualene cyclases), 233 CYPs (cytochrome P450), and 269 UGTs (UDP-glycosyltransferases). By homology-based blast and phylogenetic analysis with well-characterized genes involved in triterpenoid saponin biosynthesis, 5 *OSC*s, 14 *CYP*s, and 1 *UGT* are further proposed as the most promising candidate genes. Transcriptomic comparison between two *Ilex* species using blastp and OrthoMCL method reveals high sequence similarity. All *OSC*s and *UGT*s as well as most *CYP*s are classified as orthologous genes, while only 5 *CYP*s in *I. pubescens* and 3 *CYP*s in *I. asprella* are species-specific. One of *OSC* candidates, named as *IpAS*1, was successfully cloned and expressed in *Saccharomyces cerevisiae* INV*Sc*1. Analysis of the yeast extract by gas chromatography (GC) and gas chromatography–mass spectrometry (GC-MS) shows *IpAS*1 is a mixed amyrin synthase, producing α-amyrin and β-amyrin at ratio of 5:1, which is similar to its ortholog *IaAS*1 from *I. asprella*. This study is the first exploration to profile the transcriptome of *I. pubescens*, the generated data and gene models will facilitate further molecular studies on the physiology and metabolism in this plant. By comparative transcriptomic analysis, a series of candidate genes involved in the biosynthetic pathway of triterpenoid saponins are identified, providing new insight into their biosynthesis at transcriptome level.

## Introduction

*Ilex*, with almost 600 species, is one of the largest genera in the Aquifoliaceae family. *Ilex* species are utilized worldwide for daily consumption and health promotion. Mate tea from *I. paraguariensis* originated from the southern part of South America is now a popular health-promoting drink in western countries. Large-leaves Kudingcha from *I. kudingcha* and *I. latifolia* have been consumed as a functional food in southern China for about 2,000 years (Hao et al., 2013).

The popularity of *Ilex* and bioactive components therein lead to an increasing interest in the genetic background of these plants. With the high-throughput NGS (next-generation sequencing) technology, it is possible to depict the transcript profiling of *Ilex* species without existing genomic sequence. Recently, Debat et al. have explored the genes of *I. paraguariensis* A. St.-Hil. by NGS and *de novo* transcriptome assembly, identifying genes including those involved in different metabolic pathways and those in responses to various external stress (Debat et al., 2014). Similar studies have been carried out with *I. vomitoria* and another *I*. sp. (http://onekp.com/samples/list.php). Previously, we analyzed the transcriptome of a medicinal plant *I. asprella* using RNA-Seq, discovering several candidate genes related to the biosynthesis of triterpenoid saponins by homology alignment (Zheng et al., 2014). With the availability of more *Ilex* transcriptomes, it will surely expedite the understanding of metabolic pathway as well as evolutionary genomics and gene discovery in this interesting genus.

*I. pubescens* Hook. et Arn., a sibling plant of *I. asprella*, has long been used for the treatment of coronary heart disease, thromboangiitis obliterans and other inflammatory diseases (Zhou et al., 2008, 2013). Previous studies demonstrated that extracts of *I. pubescens* have diverse pharmacological effects, including blood vessel enlargement, anti-platelet aggregation, hypoxia-resistance, anti-inflammatory and analgesic activities (Wang et al., 2008). Like other *Ilex* species, *I. pubescene* is also characteristic for containing abundant saponins. To date, more than 70 pentacyclic triterpenoids/saponins (Table S1) have been isolated from this plant, most of which are of ursane-type (derived from α-amyrin). Triterpenoid saponins are considered as the principal bioactive components of this plant.

Triterpenoid saponins are formed by triterpenoids attached to one or more sugar moieties. Depending on their particular structures, the triterpenoids are subdivided into some 20 groups and in general lupane, oleanane and ursane tend to dominatein general. The elucidation of triterpeoid saponin biosynthesis at the molecular level has been promoted recently, because of their broad pharmacological applications (Yendo et al., 2010). However, most studies to date have been focused on lupine and oleanane type, leaving that biosynthesis of ursane-type triterpenoid saponins remains largely unknown, especially the enzymes involved in the formation of core skeleton, subsequent oxidation and glycosylation. Therefore, *I. pubescens* is fit for the study on the biosynthesis of triterpenoid saponins, in particular the ones of ursane-type.

Comparative transcriptome analysis has been widely used in the studies on biosynthesis of triterpenoid saponins. For example, the *Panax japonicus* transcriptome assembly was compared with publically available transcripts from other *Panax* species, revealing high sequence similarity across all *Panax* species and 24 CYPs (cytochrome P450) and 48 UGTs (UDP-glycosyltransferases) genes potentially involved in the downstream biosynthetic pathway of ginsenosides (Rai et al., 2016). *I. pubescens* and *I. asprella* are genetically closely related and highly similar in chemical constitutions, having 9 constituents in common (Table 1). Thus, transcriptomic comparison between these two plants may facilitate the identification of key genes in the biosynthesis of their characteristic triterpenoid saponins.

**Table 1:** **Common chemical constituents of *I. pubescens* and *I. asprella***.

Triterpenoid skeleton^*^	**Name**	**References**
B	Ursolic acid	Huang, 2011; Feng, 2012
	Pomolic acid	Han et al., 1987a; Wang, 2008
	Ilexgenin A	Hidaka et al., 1986; Zhou, M. et al., 2012
	Ilexsaponin A_1_	Hidaka et al., 1986; Zhou, M. et al., 2012
	Ziyu-glycoside I	Hidaka et al., 1986; Wang, 2008
	Ilexsaponin B_2_	Hidaka et al., 1987; Zhou, M. et al., 2012
I	Ilexodic acid	Zhang et al., 1983; Cai et al., 2010
J	Oleanolic acid	Hidaka et al., 1986; Han et al., 1987b
	Ilexasprellanosides D	Zhou, Y. et al., 2012; Lei et al., 2014

* *The triterpenoid skeleton configurations are corresponded to Figure S8*.

In this study, we performed Illumina based RNA sequencing, *de novo* assembly and functional annotation for *I. pubescens* with emphasis on the transcripts enriched in triterpenoid biosynthetic pathways. Furthermore, a comparative transcriptome analysis of *I. pubescens* with its closely related plant *I. asprella* was performed to reveal common orthologs as well as species-specific genes potentially pertaining to the biosynthesis of triterpenoid saponins. Finally, one of the orthologs encoding an oxidosqualene cyclase (named as *IpAS*1) was cloned and functionally characterized by heterologous expression in *Sacharomyces cerevisiae*. This study, therefore, might serve as a basis for the future discovery of functional genes involved in triterpenoid biosynthesis in *I. pubescens*.

## Materials and methods

### Strains and materials

*Escherichia coli* DH5α (Invitrogen, Carlsbad, CA, USA) and *S. cerevisiae* INV*Sc*1 (Invitrogen, USA) were stored and cultivated in our laboratory. α-Amyrin and β-amyrin of 98.5% purity were purchased from Sigma-Aldrich (USA). Other enzymes, unless otherwise specified, were purchased from TAKARA (Dalian, China). Medium and other chemical reagents were bought from authentic companies.

### Plant tissue collection and RNA preparation

Roots, twigs, and leaves from two wild *I. pubescens* plants grown in Pingyuan County and Panyu County of South China, respectively, were collected and snap frozen in liquid nitrogen and stored at −80°C until further processing. Total RNA was isolated using RNAiso plus and RNAiso-mate for plant tissues, following the product manual. Equal amounts of RNA from each sampled tissue were mixed to obtain a single large pool. For quality control, RNA was analyzed by using a 2100 Bioanalyzer (Agilent Technologies, Santa Clara, CA) and checked by RNase free agarose gel electrophoresis. Only those RNA with Bioanalyzer RIN value over 8 was used for cDNA synthesis.

### Library construction and sequencing

Library construction and sequencing were performed at Gene Denovo Co., Ltd., Guangzhou, China. Briefly, Poly (A) mRNA was isolated using oligo-dT beads (Qiagen, Germany). All mRNA was broken into short fragments by adding fragmentation buffer. First-strand cDNA was generated using random hexamer-primed reverse transcription, followed by second-strand cDNA synthesis using RNase H and DNA polymerase I. The double-stranded cDNA was further subjected to end reparation, a tailing and ligation to sequencing adapter. The adaptor-ligated fragments were separated on an agarose gel and a size range of cDNA fragments (200 ± 25 bp) was excised from the gel and purified. Using these purified cDNA as templates, a paired-end library was constructed using the Genomic Sample Prep kit (Illumina, USA), according to the manufacturer's instructions. Finally, the cDNA library was sequenced on the Illumina sequencing platform (Illumina HiSeq™ 2500).

### *De novo* assembly and annotation

Reads obtained from the sequencing machines included dirty reads containing adapters or low quality bases, which would affect the following assembly and analysis. Thus, to get high quality clean reads, dirty reads were filtered. Then clean reads were *de novo* assembled by the Trinity Program (Grabherr et al., 2011). The resulting UniGenes were compared with four protein databases including NCBI non-redundant database (Nr), Clusters of Orthologous Groups of protein database (COG), Swiss-Prot protein database (Swiss-Prot) and Kyoto Encyclopedia of Genes and Genomes databases (KEGG), using blastx (Altschul et al., 1997) with *E*-value cut-off of 1e^−5^. Sequence direction of the UniGenes was determined according to the best alignment results. Any UniGene that could not be aligned to the database mentioned above was then submitted to the EST Scan (http://myhits.isb-sib.ch/cgi-bin/estscan) to predict coding regions and determine sequence direction. GO annotation was analyzed by blast2GO software (https://www.blast2go.com/). Functional classification of the UniGenes was performed using WEGO software (Ye et al., 2006).

### Discovery of the genes potential involved in biosynthesis of triterpenoid saponins

#### Homology-based gene discovery

Twenty six characterized CYPs and 10 UGTs (details are shown in Table S3), which are reported to be involved in triterpenoid saponin biosynthesis, were selected as objectives to run blastp analysis against the translated sequences from the UniGenes assembled (*E*-value threshold set at 10^−5^).

#### Phylogenetic analysis

The translated sequences from UniGenes and the selected protein sequences were aligned using MUSCLE program (Edgar, 2004), and a Maximum Likelihood tree was constructed with boot strap values obtained after 1,000 replications using MEGA 7.0.14 software (Kumar et al., 2016).

### Identification of orthologous contigs and estimation of substitution rate

Orthologous of two plants were identified by blastp and OrthoMCL method setting the *E*-value cutoff at 1E-10. Genes with the *E*-value less than 1E-10 are thought as orthologs and the others are specific genes of each species. Then orthologs or specific genes were classified into different families by OrthoMCL method. All kinds of genes and gene family numbers were counted.

The ratio of the number of non-synonymous substitutions pernonsynonymous site (Ka) to the number of synonymous substitutions per synonymous site (Ks) was used to test for positive selection. The rate of Ka to Ks between putatively orthologous coding regions were estimated based on the maximum-likelihood method (Yang and Nielsen, 2000) using KaKs_Calculator 2.0 with the YN model (Wang et al., 2010). The orthologs with Ks rate < 0.1 were excluded from further analysis to avoid inclusion of paralogs (Elmer et al., 2010).

Based on the Ka/Ks value with the threshold set at 0.5, the orthologs were subcategorized into two datasets: a test set with Ka/Ks > 0.5, and a reference dataset with Ka/Ks value < 0.5. The significance of the difference in GO term abundance between the two datasets was tested using the Fisher's exact test with the GOSSIP package (Blüthgen et al., 2005) implemented in blast2GO V.2.6.0 (Conesa et al., 2005).

### Characterization of a mixed amyrin synthase (AS)

#### cDNA preparation and cloning of AS gene

Total RNA was extracted as described above. Polyadenylated RNA was isolated and translated into cDNA using oligo (dT) primers by following the protocol of the FastQuant RT Kit (Tiangen, Beijing, China). The cDNA served as a template for the amplification of *IpAS*1 using high fidelity DNA polymerase with one set of gene-specific primer (IpAS1-F and IpAS1-R1, see Table S4) under the following cycling conditions: 98°C for 2 min; 30 cycles of 98°C for 10 s, 55°C for 15 s, 72°C for 15 s; and 72°C for 5 min. The resulting PCR product was directly ligated into the pLB vector using Zero Background Fast Cloning Kit (Tiangen, Beijing, China), transformed into *E. coli* DH5α and submitted for sequencing. The obtained plasmid was named as pLB-AS1.

#### Construction of expression plasmids

The CDS of *IpAS*1 was amplified from the plasmid pLB-AS1 using the primer pair IpAS1-F and IpAS1-R2 (see Table S4). The PCR product was sub-cloned into pESC-URA vector (Agilent, USA) by using infusion cloning technology, resulting in the plasmid pESC-U-AS1. pESC-U-AS1 was transformed into *S. cerevisiae* INV*Sc*1 using a standard lithium acetate protocol (Gietz and Woods, 2002), with the empty pESC-URA as the negative control.

#### Protein expression

*S. cerevisiae* INV*Sc*1 containing pESC-U-AS1 was grown in SC-U media containing 2% raffinose for overnight. The cells were collected and resuspended in SC-U media containing 2% galactose for induction. And then the cells were harvested at different time points over a period of 16 h and extracted for total proteins. The target protein was identified using HIS mouse monoclonal antibody (Santa Cruz, CA, USA) by Western blotting.

#### GC and GC-MS analysis

Yeast cells after induction for 72 h were collected and refluxed with 20 mL extracting solution [20% KOH (m/v)/50% EtOH (v/v)] for 30 min, then extracted with n-hexane for twice. Extracts (n-hexane layer) were evaporated to dryness, resuspended in methanol and submitted to GC (gas chromatography) and GC-MS (gas chromatography-mass spectrometry) analysis directly. GC analysis was performed on an Agilent 7980B GC machine equipped with a flame ionization detector (FID) and a HP-5MS column (30 m × 0.25 mm × 0.25 μm, Agilent, CA, USA). The column temperature was set at 80°C for 1 min, followed by a 20°C/min ramp to 200°C, followed by a 10°C /min ramp to 310°C, held at 310°C for 15 min. Injector and detector temperatures were both set at 250°C. The sample was injected in a splitless injection mode and the carrier gas was helium with a flow rate of 1.2 mL/min. GC-MS was performed on an Agilent 7980B-5977A GC/MSD machine, the column and gas phase temperature program were same as GC analysis method mentioned above. And the injector was set at a 10:1 split stream mode, with a temperature of 250°C. The flow rate of helium carrier gas was 0.70 mL/min. Ionization of samples was performed by electron impact at 70 eV and temperature at 230°C, The data were acquired over a mass range of *m*/*z* 29–600.

## Results

### *De novo* assembly and functional annotation of *I. pubescens* transcriptome

After cleaning of raw sequences, 49,084,824 high quality (HQ) reads were obtained with the Q20 and GC percentages of 96.84 and 44.42%, respectively. The HD clean reads have been uploaded to the Sequence Read Archive (SRA) at NCBI with the accession number SRP102344. *De novo* assembly of these HQ reads produced 68,688 UniGenes of 6,135,603,000 nucleotides (nt). The average length of these UniGenes was 746 nt, with an N50 of 1,333 nt. The length distribution of *I. pubescens* unigenes was shown in Figure S1.

UniGenes were successfully annotated through comparison with sequences in the major public databases, such as Nr, COG, Swiss-Prot and KEGG. A total of 32,184 UniGenes had at least one significant match with an *E*-value less than 1e^−5^ against four databases Figure S2, which accounted for 46.86% (Table 2). Out of the annotated UniGenes, 5,128 are common among the four databases, 9,821 are matched uniquely in Nr database and 140 found hits only in Swiss-Prot (Figure S3). There are also 14 and 1 UniGenes annotated uniquely by KEGG and COG, respectively. Many identified genes showed significant similarity to those from *Vitis vinifera* (11.89% of total UniGenes), *Theobroma cacao* (7.93%) and *Solanum lycopersicum* (5.46%) (Figure S4).

**Table 2:** **UniGenes mapped to the public databases**.

**Public database**	**No. of matched UniGenes**	**Annotation percentage (%)**
Nr	31,994	46.58
Swiss-Prot	21,508	31.31
KEGG	8,386	12.21
COG	9,854	14.35
Total	32,184	46.86

For functional prediction and classification against the COG database, 8,386 UniGenes were grouped into 25 COG classifications. The cluster for “general function prediction only” represented the largest group (2,624 UniGenes), followed by “replication, recombination and repair” (1,409 UniGenes), “posttranslational modification, protein turnover, catabolism” (1,325 UniGenes) and “transcription” (1,293 UniGenes). 424 UniGenes were assigned to the cluster “secondary metabolites biosynthesis, transport and catabolism” (Figure S5).

For biochemical pathways prediction in the KEGG database, 8,386 UniGenes were mapped to 124 KEGG pathways. In these 124 pathways, 2,223 UniGenes were mapped to “Metabolic pathways” (pathway ID Ko01100), followed by “Biosynthesis of the secondary metabolites” (pathway ID Ko01110, 1,076 UniGenes) and “Ribosome” (pathway ID Ko03010, 651 UniGenes). Out of these, 128 UniGenes were assigned triterpenoids biosynthesis processes. 63 UniGenes (0.75%) were mapped to “Terpenoid backbone biosynthesis,” 4 (0.05%) were mapped to “Monoterpenoid biosynthesis,” 14 (0.17%) were mapped to “Diterpenoid biosynthesis,” 6 (0.07%) were mapped to “Sesquiterpenoid and triterpenoid biosynthesis” and 41 (0.49%) were mapped to “Ubiquinone and other terpenoid-quinone biosynthesis.” Subsequently, candidate genes related to terpenoid backbone and triterpenoid synthesis were identified and discussed in detail.

Gene Ontology (GO) assignments were used to classify the functions of all UniGenes. Based on sequence homology, 15,610 UniGenes were mapped to 67 functional groups, which were distributed under three main categories including biological processes (11,244 UniGenes), cellular components (12,502 UniGenes) and molecular functions (8,715 UniGenes). From the biological process class, 8,231 UniGenes were involved in the “metabolic process” (Figure S6).

### Enrichment of triterpenoid biosynthetic pathways

Terpenoids are built up from C5 units, isopentenyl diphosphate (IPP), which is supplied either from the cytosolic mevalonate pathway (MVA pathway) or from the plastidal methylerythritol phosphate pathway (MEP pathway). Triterpenoids are biosynthesized via MVA pathway (Figure S7). Due to the biological importance of sterol and diterpenoid, the previous steps in its conversion from acetyl-CoA and 1-deoxy-D-xylulose-5-phosphate to IPP have been widely studied in many plant species, but the following steps remained unclear, especially the late steps of the pathways. The cyclization of oxidosqualene catalyzed by oxidosqualene cyclase (OSCs, EC 5.4.99.x) is the branch point for the biosynthesis of triterpenoid and sterol. According to the proposed pathways, some specific cytochrome P450s (CYPs, EC 1.14.x.x) and UDP-glycosyltransferases (UGTs, EC 2.4.1.x) (family 1 uridine diphosphate glycosyltransferases) may catalyze various triterpenoids (Tang et al., 2011; Seki et al., 2015).

**Figure 1:**
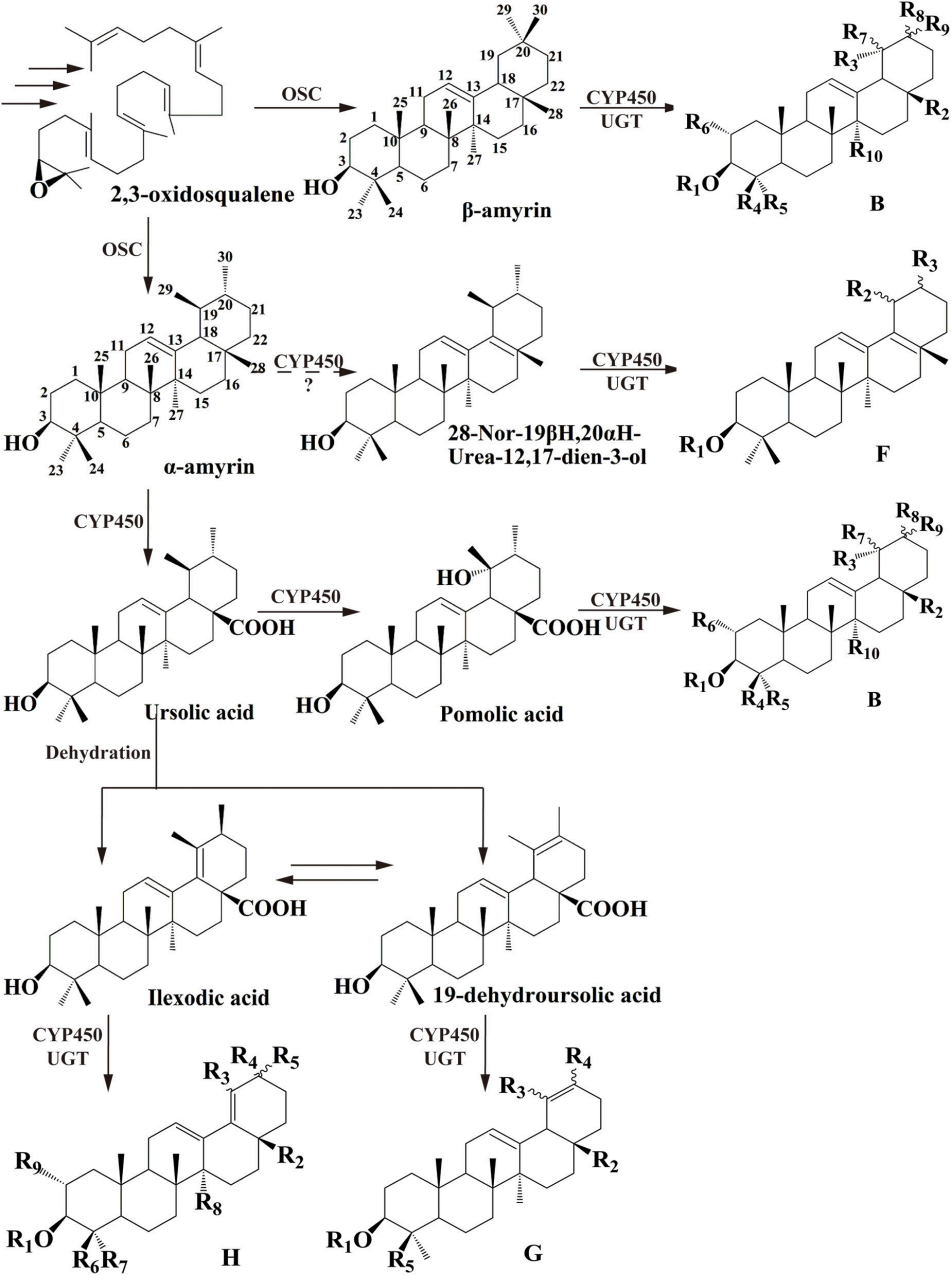
**Proposed downstream biosynthetic pathway of triterpenoid saponins of common structures in *I. pubescens* and *I. asprella***. The structure numbers were corresponding to Figure S8.

Among dozens of pentacyclic triterpenoids isolated from *I. pubescens* and *I. asprella* (see Tables S1, S2), there are 9 different skeleton structures. Structure B, F, G, and H therein are common for both species. While structure D and E are unique from *I. pubescens*, structure A, C, and I exist only in *I. asprella* (Figure S8). Base on the chemical structures, a putative biosynthetic pathway of the triterpenoids of common structures from both species are proposed and the expected key enzymes are deduced, as shown in Figure 1 and Table 3, respectively.

**Table 3:** **Expected key enzymes of triterpenoid biosynthesis in *I. pubescens* and *I. asprella***.

	Structural locus^*^	***I. asprella***	***I. pubescens***	**Key enzymes**
Nucleus	α-amyrane, β-amyrane	√	√	OSC (α-AS, β-AS, multifunction-AS)
Double bond	17 (18), 18 (19), 19 (20)	√	√	CYP (?)
	14 (15), 20 (21)	–	√	
	19 (29)	√	–	
Hydroxylation	19, 27	√	√	CYP
	20, 23, 24	–	√	
	2	√	–	
Carboxylation	23, 24, 28	√	√	
Aldehydes	23	–	√	
Glycosylation	3, 28	√	√	UGT
Sulfonation	3	√	√	Sulfo transferase

* *The structural numbers were corresponding to Figure S8*.

#### Terpenoid backbone biosynthesis of *I. pubescens* transcriptome

The MVA pathway is essential for the biosynthesis of sterols, sesquiterpenes and triterpenoids. 16 UniGenes in this transcriptome, including 2 AACT (acetyl-CoA acyltransferase, EC 2.3.1.9) genes, 4 HMGS (3-hydroxy-3-methylglutaryl-CoA synthase, EC 2.3.3.10) genes, 6 HMGR (3-hydroxy-3-methylglutaryl-CoA reductase, EC 1.1.1.34) genes, 1 MK (mevalonate kinase, EC 2.7.1.36) gene, 1 PMK (phosphomevalonate kinase, EC 2.7.4.2) gene and 2 MDC (mevalonate 5-dinhophate decarboxylase, EC 4.1.1.33) genes were identified to be involved in this pathway.

Monoterpenes and diterpenes are synthesized through the MEP pathway. 18 UniGenes encoding enzymes involved in this pathway of *I. pubescens* transcriptome, including 6 DXS (1-deoxy-D-xylulose 5-phosphate synthase, EC 2.2.1.7) genes, 4 DXR (1-deoxy-D-xylulose 5-phosphate reductoisomerase, EC 1.1.1.267) genes, 2 MCT (MEP cytidyltransferase, EC 2.7.7.60) genes, 3 HDR (4-hydroxy-3-methylbut-2-enyldiphosphate reductase, EC 1.17.1.2) genes and 1 each of CMK (4-(Cytidine 5-diphospho)-2-C-methylerythritol kinase, EC 2.7.1.148), MDS (2-C-Methy-D-erythritol 2,4-cyclodiphosphate synthase, EC 4.6.1.12) and HDS (hydroxymethylbutenyl 4-diphosphate synthase, EC 1.17.7.1).

Both MVA and MEP pathways produce the C5 unit IPP, which can be transformed into its isomer, DMAPP (dimethylallyl diphosphate) by IDI (isopentenyl diphosphate isomerase, EC 5.3.3.2). Meanwhile, IPP and DMAPP are assembled into GPP (geranyldiphosphate), FPP (diphosphate) and GGPP (geranylgeranyl diphosphate) by a series of prenyltransferases, including GPPS (geranyl diphosphatesynthase), FPPS (farnesyl diphosphate synthase, EC 2.5.1.1) and GGPPS (geranylgeranyl diphosphate synthase, EC 2.5.1.10). FPP is an important intermediate of triterpenoid biosynthesis. Two units of FPP join in a “tail-to-tail” fashion, catalyzed by squalene synthase (SS, EC 2.5.1.21), to yield the hydrocarbon squalene. Then subsequently, squalene is oxidized by squalene monooxygenase (SM, EC 1.14.13.132) with the cofactors O_2_and NADPH (nicotinamide adenine dinucleotide phosphate) to give rise to another important precursor, 2,3-oxidosqualene (Haralampidis et al., 2002; Vincken et al., 2007). In our study, 2 IDI genes, 3 GPPS (geranylpyrophosphate synthase, EC 2.5.1.1) genes, 2 FPPS genes, 5 GGPPS genes, 2 SS genes and 4 SM genes were annotated in this transcriptome.

#### Triterpenoid downstreams biosynthesis of *I. pubescens* transcriptome

**Figure 2:**
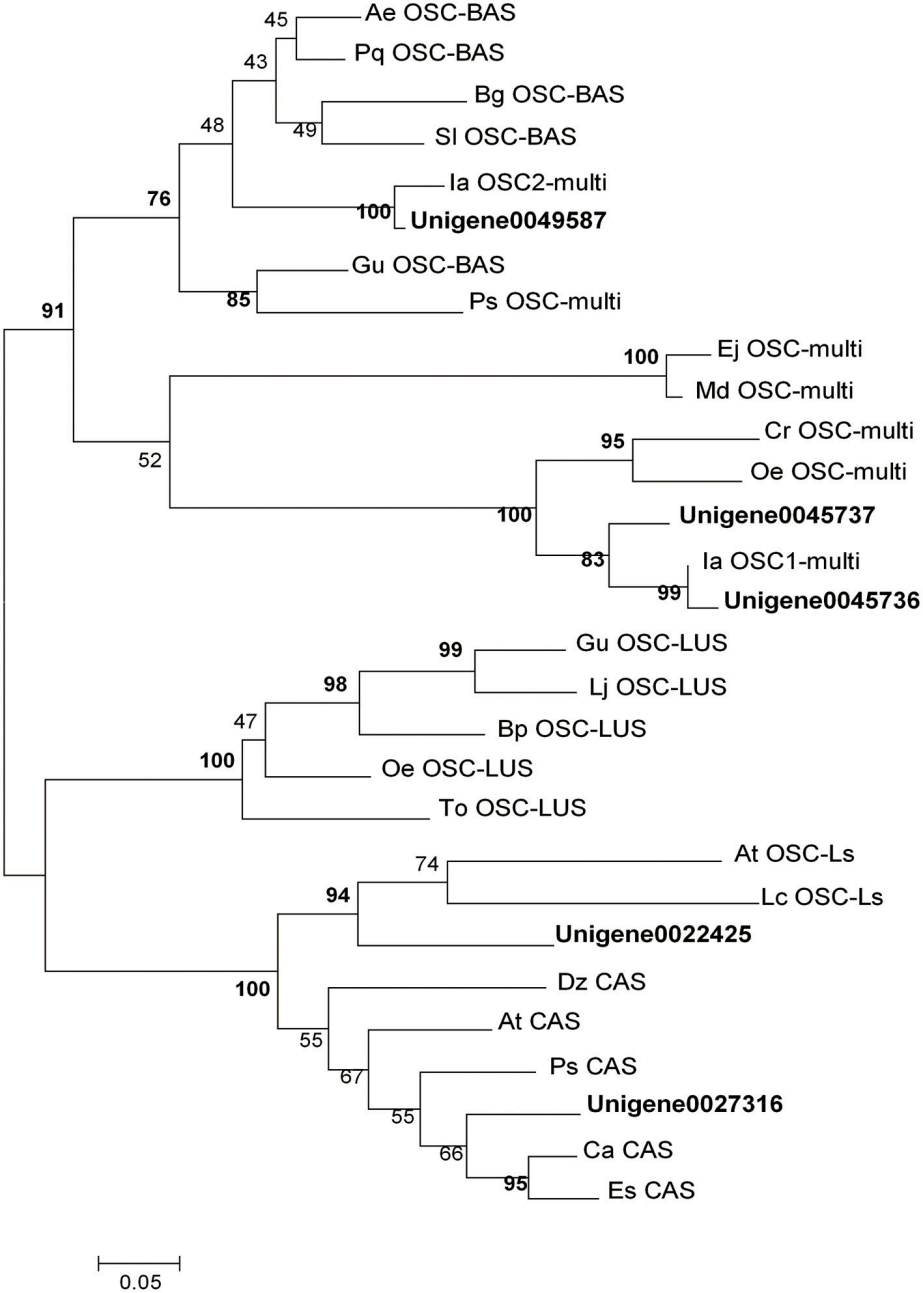
**Phylogenetic analysis of OSCs**. The statistical methods of this unrooted tree was maximum likehood method, which was tested by bootstrap method and the number of bootstrap replications was 1,000. The bold indicate the OSCs were selected from *I. pubescens* transcriptome and the others were randomly selected from GenBank. Ae, *Aralia elata;* At, *Arabidopsis thaliana*; Bp, *Betula platyphylla*; Ca, *Cicer arietinum*; Cr, *Catharanthus roseus*; Dz, *Dioscorea zingiberensis*; Ej, *Eriobotrya japonica*; Es, *Eleutherococcus senticosus*; Gu, *Glycyrrhiza uralensis*; Ia, *Ilex asprella*; Lc, *Luffa cylindrica*; Lj, *Lotus japonicus*; Md, *Malus domestica*; Oe, *Olea europaea*; Pq, *Panax quinquefolius*; Ps, *Pisum sativum*; Sl, *Solanum lycopersicum*; To, *Taraxacum officinale*. BAS, β-amyrin Synthase; CAS, cycloartenol synthase; LUS, lupeol synthase; LS, lanosterol synthase; Multi, multifunctional OSC gene.

As previously described, OSCs catalyze the cyclisation of 2,3-oxidosqualene to form a variety of triterpene skeletons (Cordoba et al., 2011), including phytosterol, dammarane, lupane and oleane (β-amyrin). This step is thus a critical branching point for phytosterol and triterpenoid biosynthesis. In this study, 11 UniGenes were identified to be amyrin synthase (abbreviated as AS) genes. Among them, 5 UniGenes were longer than 1,000 bp. Phylogenetic analysis of the translated sequences of these 5 UniGenes with 24 characterized OSCs randomly selected from GenBank was carried out. UniGene0045736 (named as IpAS1), UniGene0045737 and UniGene0049589 are high-homology with those identified *OSC*s (*IaAS*1 and *IaAS*2) from *I. asprella*, implying they may have the same functions (Zheng et al., 2015). Moreover, UniGene0022425 and UniGene0027316 were annotated to be lanosterol synthase and cycloartenol synthase, respectively (Figure 2). Among these 5 candidate UniGenes, *IpAS*1 (PKRM = 103.31) was found to contain a full-length cDNA, including start and stop codons and a polyA signal, using the online tool GENSCAN (http://genes.mit.edu/GENSCAN.html) and ORF Finder (http://www.ncbi.nlm.nih.gov/projects/gorf/). Thus, IpAS1 was further cloned and functionally characterized.

Following the formation of hydrocarbon skeleton, functional groups, like hydroxyl group and carboxyl group are introduced at different positions of the backbone, which is supposed to be catalyzed by CYPs. CYP family is one of the largest and most diverse gene families in plants. Up to now, more than 20 *CYPs* were identified in pentacyclic triterpenoids biosynthesis (Shibuya et al., 2006; Seki et al., 2008; Huang et al., 2012; Fukushima et al., 2013; Geisler et al., 2013; Guo et al., 2013; Han et al., 2013; Moses et al., 2014a,b; Moses et al., 2015a,b; Yasumoto et al., 2016). Phylogenetic analysis of these *CYPs* was showed in Figure S9, which suggests that the members in CYP71 clan may act as C24 oxidase, while in CYP72 clan and CYP85 clan may exist multi-functional enzymes in pentacyclic triterpenoids biosynthesis. In the transcriptome of *I. pubescen*s, 233 UniGenes were annotated as CYPs and their characteristics were shown in Figure S10. The *CYP*s with gene length >1,000 bp and classified to CYP72 clan and CYP85 clan (identity >55% means in the same subfamily of *CYP*s, Nelson, 2011) were shown in Table S5 (14 Unigenes), which are the most promising candidate genes and may include new genes with new functions.

UGTs catalyze the transfer of glycosyl residues to triterpenoids that are decorated by CYPs, increasing aqueous solubility and making them into triterpenoid saponins. The glucosylation of C3-hydroxyl and C28-carboxyl is observed in a number of triterpenoid saponins in *I. pubescens*. Like CYPs, UGTs constitute a large and diverse gene family. Sequences belonging to the same family and subfamily exhibit amino acid sequences identity >40% and >60% (Mackenzie et al., 1997; Augustin et al., 2011), respectively. Up to now, about 10 *UGT*s (Meesapyodsuk et al., 2007; Naoumkina et al., 2010; Shibuya et al., 2010; Augustin et al., 2012; Sayama et al., 2012; Yan et al., 2014) were identified in pentacyclic triterpenoids biosynthesis. Phylogenetic analysis of these *UGT*s were shown in Figure S11, indicating that UGT73 clan may exist multi-functional enzymes. In the transcriptome of *I. pubescens*, 269 UniGenes were found to encode UGTs. Among them, only 1 UniGene exhibited high homology (85.71%) to UGT73C10 and UGT73C12 from *Barbarea vulgaris* (Augustin et al., 2012), which catalyze the 3-*O*-glucosylation of oleanolic acid.

### Comparative transcriptomic analysis

#### Gene protein family, orthologous contigs, substitution rates, and transcriptome divergence between two *Ilex* species

Coding sequences from *I. pubescens* and *I. asprella* transcriptomes were used to carry out comparative analysis. Thus, a total of 33,972 UniGenes belonging to 12,756 gene families were classified as orthologs, much more than specific genes of *I. pubescens* (3,423 UniGenes, 836 gene families) or *I. asprella* (2,829 UniGenes, 1,183 gene families) (Figure S12). Based on functional classification by KEGG, candidate genes related to terpenoid backbone were all clustered to common genes.

After removing the sequences with Ks > 0.1 and sequences with all non-synonymous substitutions or synonymous substitutions, 7,635 unique orthologs were left, with the mean values of Ka, Ks, and Ka/Ks ratio as 0.016, 0.0051, and 1.37, respectively. Of these, 582 orthologs had a Ka/Ks ratio >1.0, and 1,154 ortholog pairs had a Ka/Ks ratio between 0.5 and 1.0 (Figure S13). These genes with Ka/Ks ratio significantly higher than 1 likely experienced diversifying selection, with which the amino acid change may offer a selective advantage (Yang and Bielawski, 2000). Ka/Ks ratio > 0.5 is a less conservative cut-off, but it has also been proven useful for identifying genes under positive selection (Elmer et al., 2010). Adaptive molecular evolution in most convincing cases has been identified through the Ka/Ks ratio in protein-coding DNA sequences (Yang and Bielawski, 2000). Therefore, all of these 1,736 orthologs (Ka/Ks > 0.5) were considered as candidate genes that have probably experienced positive selection.

#### Enrichment of triterpenoid biosynthetic pathways by comparative transcriptome

The key enzymes genes involved in the upstream of triterpenoids biosynthesis (included MVA pathway, MEP pathway and their middle pathway, see KEGG map 00900 and Figure S7) of *I. pubescens* and *I. asprella* transcriptomes were annotated as orthologs, except for 4 HMGR genes in *I. asprella* noted as specific genes. *OSC*s (8 UniGenes), *CYP*s (252 UniGenes) and *UGT*s (100 UniGenes) were clustered as orthologs of *I. pubescens* and *I. asprella*. At the same time, 5 *CYP*s in *I. pubescens* and 3 *CYP*s in *I. asprella* were designated as species-specific genes.

In the orthologs of 8 *OSC*s in *I. pubescens* and *I. asprella* transcriptomes, 4 UniGenes were from *I. pubescens* transcriptome and 4 UniGenes from *I. asprella* transcriptome (see Table 4). Among them, CL3079_Contig1 (named as *IaAS*1) and CL481_Contig1 (named as *IaAS*2) in *I. asprella* transcriptome are multifunctional amyrin synthase, catalyzing the formation of α-amyrin and β-amyrin at the ratio of 4:1 and 1:19, respectively (Zheng et al., 2015). Comparative transcriptomic analysis revealed UniGene0045736 in *I. pubescens* transcriptome is an ortholog of IaAS1 and it may also be a multifunctional amyrin synthase with α-amyrin as major product.

**Table 4:** **Orthologs of *OSC*s in *I. pubescens* and *I. asprella* transcriptomes**.

***I. pubescens***		***I. asprella***	
**UniGene**	**Length**	**UniGene**	**Length**
UniGene0061368	487	UniGene1015	993
UniGene0049587	3,401	CL481_Contig1	2,892
UniGene0045736	3,948	CL3079_Contig1	2,707
UniGene0045737	1,763	CL481_Contig2	2,968

**Figure 3:**
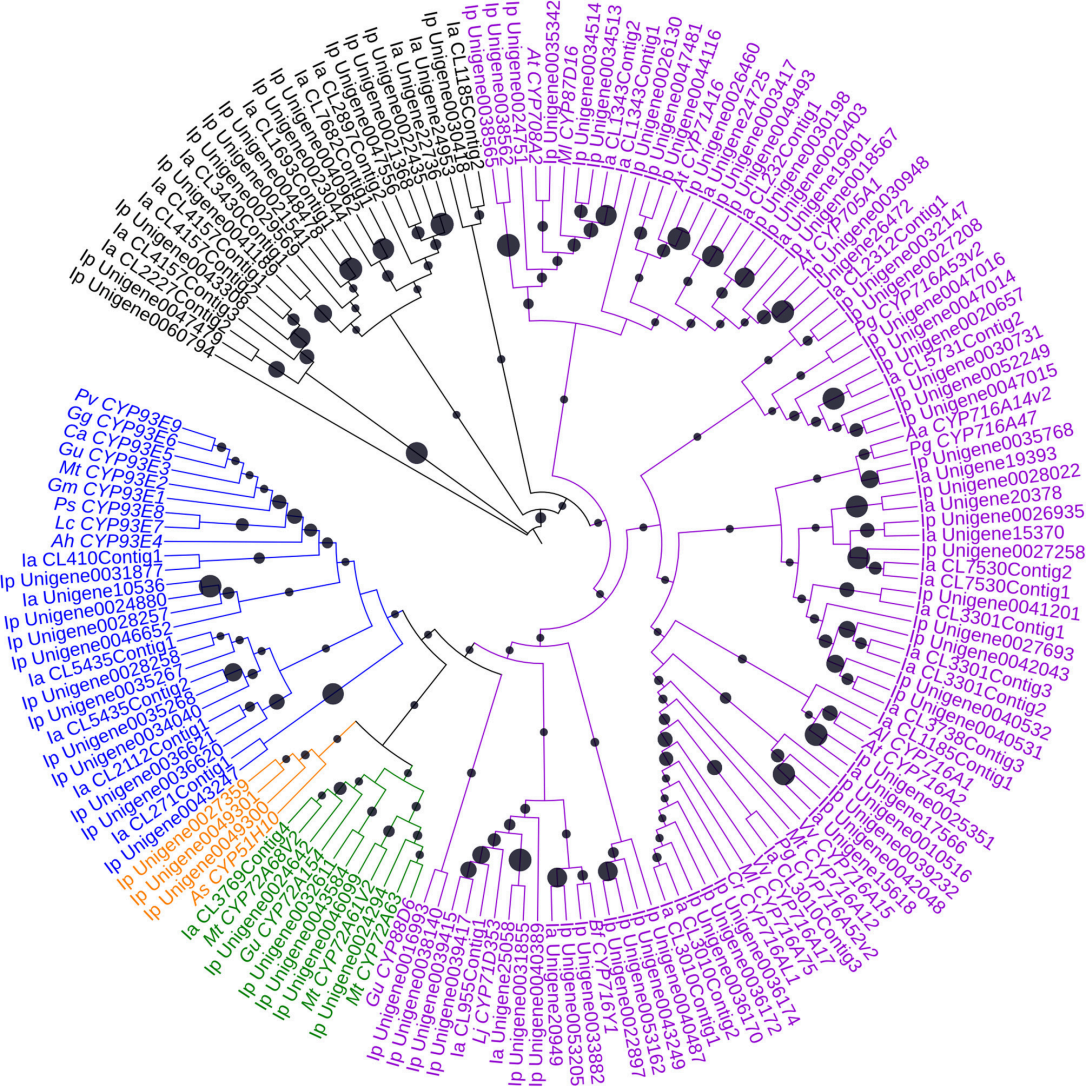
**Phylogenetic analysis of CYPs**. The statistical methods of this unrooted tree was the same as Figure 2. The blue, orange, green, purple and black indicate the indicate the cytochrome P450 clustered together with the CYP71, CYP51, CYP72, CYP85 and other clans, respectively. Black circle size means the bootstrap percentage of total number test. Aa, *Artemisia annua*; Ah, *Arachis hypogaea*; Al, *Arabidopsislyrata* subsp. *lyrata*; As, *Avena strigosa*; At, *Arabidopsis thaliana*; Bf, *Bupleurumfalcatum*; Ca, *Cicer arietinum*; Cr, *Catharanthus roseus*; Gg, *Glycyrrhiza glabra*; Gm, *Glycine max*; Gu, *Glycyrrhiza uralensis*; Ia, *Ilex asprella*; Ip, *Ilex pubescens*; Lc, *Lens culinaris*; Ml, *Maesa lanceolata*; Mt, *Medicago truncatula*; Pg, *Panax ginseng*; Ps, *Pisum sativum*; Pv, *Phaseolus vulgaris*; Vv, *Vitis vinifera*.

Of the orthologs of 252 *CYP*s in *I. pubescens* and *I. asprella* transcriptomes, 139 UniGenes were from *I. pubescens* transcriptome and 113 UniGenes from *I. asprella* transcriptome. Furthermore, these *CYP*s were submitted to the database of essential genes (http://www.essentialgene.org/) to compare with essential genes of *Arabidopsis thaliana*. In the result, 73 of 139 UniGenes in *I. pubescens* transcriptome and 60 of 113 UniGenes in *I. asprella* transcriptome could be homologous with essential genes with *A. thaliana*. And a phylogenetic analysis was performed with UniGenes the length greater than 1,000 bp of these 252 UniGenes to identify *CYP*s involved in triterpenoid biosynthesis (Figure 3).

To further screen the genes potential related to triterpenoid saponin biosynthesis from *I. pubescens* and *I. asprella* transcriptomes, comparative analysis with a diterpene producer was carried out. Phylogenetic analysis of the well characterized *CYP*s involved in diterpenoid biosynthesis and those involved in triterpenoid biosynthesis displayed that they could be separated from each other and cluster to different branches in phylogenetic tree (Figure S14). *Andrographis paniculate* (Burm.f.) Nees is an *Acanthaceae* plant, which contains mostly diterpenoids and flavonoids, no triterpenoids have been found until now. Thus, comparative analysis was taken between *Andrographis paniculate* transcriptome (unpublished data) and the orthologs of *CYP*s in *I. pubescens* and *I. asprella* transcriptomes. As a result, 77 *CYP* UniGenes were unique in *I. pubescens* and *I. asprella* transcriptomes, which may involve in triterpenoid biosynthesis. Of them, 43 UniGenes were in *I. pubescens* transcriptome and 34 UniGenes were from *I. asprella* transcriptome (Table S6).

The orthologs of 100 *UGT*s in *I. pubescens* and *I. asprella* transcriptomes consist of 50 UniGenes in *I. pubescens* transcriptome and 50 Unigenes in *I. asprella* transcriptome. These *UGT*s were also submitted to database of essential genes (http://www.essentialgene.org/) to compare with essential genes of *Arabidopsis thaliana*. We did not found any genes homologous with essential genes of *Arabidopsis thaliana*.

### Functional characterization of a mixed amyrin synthase

#### Isolation and sequence analysis of IpAS1

To confirm the reliability of the transcriptomic data, *IpAS*1 was subjected to functional characterization. The full-length cDNA of *IpAS*1 was successfully isolated from the *I. pubescens* cDNA library by using the designed primers beyond the open reading frame (ORF), which encodes a protein of 762 amino acids with a mass of 87.6 kDa. *IpAS*1 shares 98% sequence similarity to *IaAS1* (mixed amyrin synthase gene, *I. asprella*, GI: AIS39793.1), 82% sequence similarity to *CrAS1* (mixed amyrin synthase, *Catharanthus roseus*, GI: AFJ19235.1) and 62% sequence similarity to *MdOSC1* (mixed amyrin synthase, *Malus domestica*, GI: ACM89977.1). As shown in Figure S15, six QW motifs, a DCTAE motif and a MWCYCR motif were found in the protein sequences of *IpAS*1. QW motifs were believed to be responsible for strengthening the structure of the enzyme and stabilizing its carbocation intermediates (Poralla et al., 1994; Wendt et al., 1999; Kushiro et al., 2000) and DCTAE motifs may play an important role in substrate binding (Abe and Prestwich, 1994). In addition, MWCYCR motifs may be related to the product specificity of β-amyrin synthase (Kushiro et al., 2000), respectively.

#### Gene cloning and protein expression

**Figure 4:**
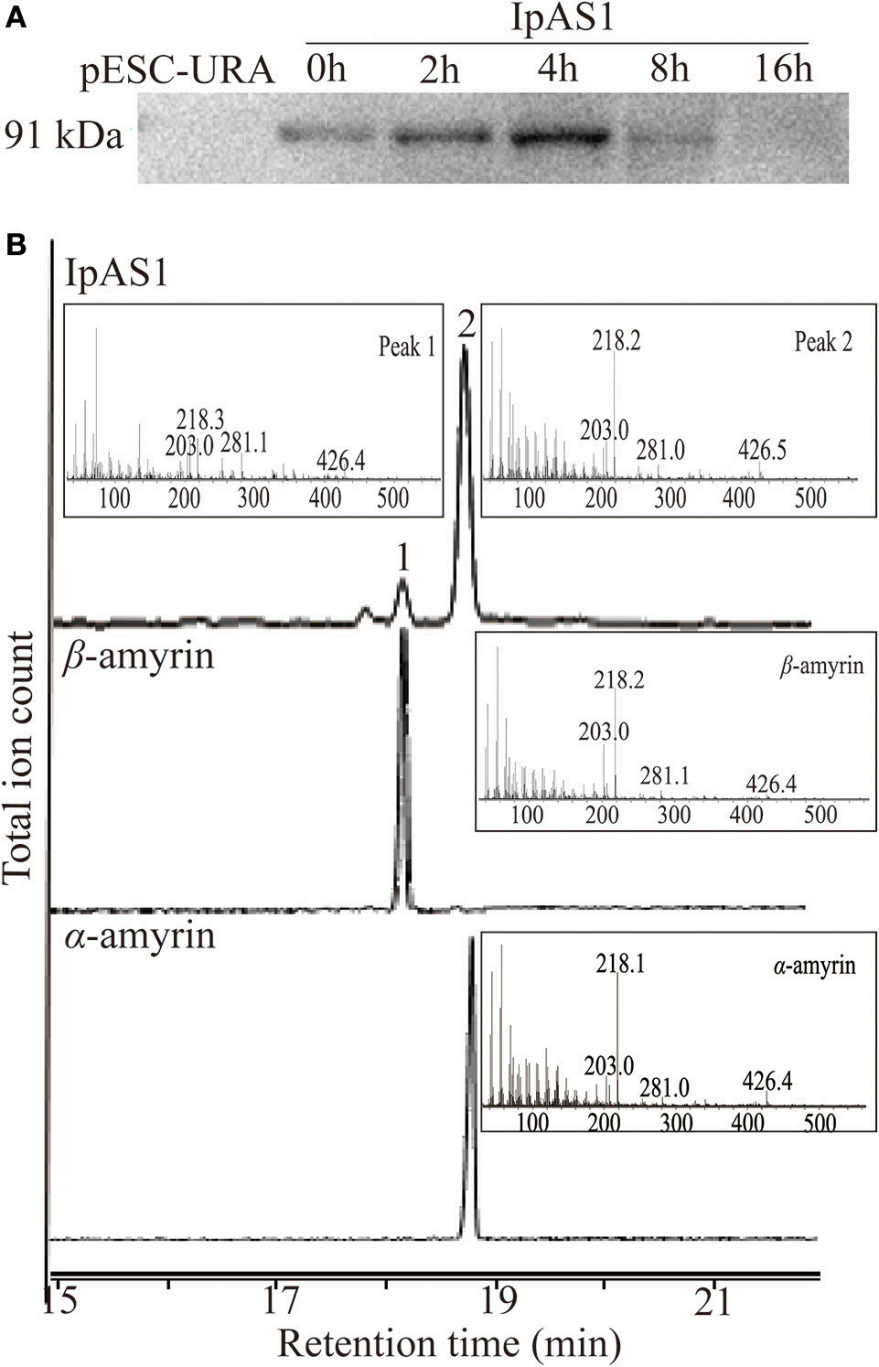
**Functional identification of *IpAS*1. (A)** Western blot analysis of *IpAS*1 expressed in *S. cerevisiae* INV*sc*1. Equal amount of yeast cells were harvested at five different time points during 16 h of induction by Gal (0, 2, 4, 8, and 16 h), which showed that *IpAS*1 was successfully expressed during the whole induction, with a maximum band intensity observed at 4 h. **(B)** GC-MS analysis of yeast metabolites after expression of *IpAS*1. By comparing the retention times and mass fragment patterns with authentic standards, two compounds were identified as α- and β-amyrin, respectively, which indicates IpAS1 is a mixed AS.

To elucidate the enzymatic activities of *IpAS*1, the ORF of this gene was cloned into yeast expression vector pESC-URA under the control of galactose (Gal) promoter. Then the construct was transformed into *S. cerevisiae* INV*Sc*1, which synthesizes 2, 3-oxidosqualene endogenously. Equal amount of yeast cells were harvested at five different time points during 16 h of induction by Gal. Western blotting analysis of the total protein extracted from the cells showed that *IpAS*1 was successfully expressed during 16 h of induction, with a maximum band intensity observed at 4 h (see Figure 4A).

#### Functional analysis of IpAS1 in yeast

Triterpene products were extracted after 72 h of induction and analyzed by GC and GC-MS. The extracts of yeast carrying *IpAS*1 contained two more compounds, as compared with negative control (yeast carrying the empty vector). Two compounds were identified as α- and β-amyrin, respectively, by comparing their retention times and mass fragment patterns with authentic standards. Therefore, *IpAS*1 is a mixed AS (see Figure 4B), producing α- and β-amyrin at a ratio of above 5:1. Compared with other mixed ASs, *IpAS*1 exhibits a unique product specificity toward α-amyrin, even higher than *MdOSC1* in *Malus* × *domestica*, the AS with the highest rate *of* α-amyrin production reported up to date (Brendolise et al., 2011). To verify the results, different transformants of *IpAS*1 was analyzed in parallel. As shown in Table 5, the ratio of the two compounds of different transformants was reproducible, more than 5:1.

**Table 5:** **Productions of α-amyrin, β-amyrin by the different Yeast transformant carrying *IpAS*1 (*n* = 2)**.

**Strain**	**Repeat**	**Peak area of β-amyrin**	**Peak area of α-amyrin**	**Peak area of α-amyrin/Peak area of β-amyrin**
No.1	1	536.95	2, 869.40	5.34
	2	517.65	2, 681.55	5.18
No.2	1	93.15	508.10	5.45
	2	95.00	505.30	5.32
No.3	1	508.30	2, 628.40	5.17
	2	436.50	2, 232.66	5.11

## Discussions

Triterpenoid saponins are known to be synthesized via the isoprenoid pathway by cyclisation of 2, 3-oxidosqualene to give primary skeleton. The triterpenoid backbone then undergoes various modifications (oxidation, substitution, and glycosylation) (Haralampidis et al., 2002). However, the triterpenoids synthases are less abundant in a particular organ or structure, or have low sequence similarity to known triterpenoids synthases (Bleeker et al., 2011). Meanwhile, CYPs and UGTs were divergent, polyphyletic and multigene families (Paquette et al., 2003). All these limit the study of triterpenoid saponin biosynthesis. RNA-Seq, a next generation sequencing technology, is being used as one of the most efficient tools for gene discovery and various functional studies (Jayakodi et al., 2014). Illumina transcriptome sequencing and assembly have been used successfully for gene discovery in terpenoid saponin biosynthesis, such as artemisinin (sesquiterpene), taxol (diterpene), ginsenoside (triterpene). Similarly, RNA-seq of *I. pubescens* was used to discover genes involved in triterpenoids biosynthesis in this study. To effectively identify candidate genes, comparative transcriptomic analysis together with structural characteristics of triterpenoids (including similarities and differences) of *I. pubescens* and *I. asprella* were used in this study. As a result, the candidates of *CYP*s and *UGT*s was narrowed down significantly (about 5-fold). Comparison of transcripts linked to metabolite profiling has been used for gene discovery in triterpenoid biosynthesis in *panax* genus (Chen et al., 2011; Rai et al., 2016) or monoterpene biosynthesis in *Stevia* genus (Chen et al., 2014). Therefore, comparative analysis of transcriptomes of different species within a same genus must be a useful tool to study the metabolic pathway.

Many species of the *Ilex* genus plants are rich in triterpenoid saponins, mostly of ursane-type (derivative of α-amyrin) (Zheng et al., 2014). Study of pentacyclic triterpenoid biosynthesis in Aquifoliaceae has been undertaken in *I. aquifolium* (Niemann, 1985) and *I. Asprella* (Zheng et al., 2014). The investigation to elucidate the biosynthetic mechanism of triterpenoid saponins in *I. pubescens* will contribute to the understanding of the metabolism of these important plant. Functional characterization of *IpAS*1 showed that *IpAS*1 is a mixed α-amyrin synthase, producing mainly α-amyrin, which is consistent with that *I. pubescens* contains largely ursane-type triterpenoid saponins. 5 *CYP*s in *I. pubescens* and 3 *CYP*s in *I. asprella* were classed as specific genes, implying they may be related to the formation of triterpenoids of structure D and E in *I. pubescens* and of structure A, C and I in *I. asprella*, respectively. Among 79 UniGenes of *CYP*s identified as orthologous genes between *I. pubescens* and *I. asprella*, there must be genes potential involved in the biosynthesis of triterpenoids of structure B, F, G, and H. As far as *UGT*s, only a 3-*O*-glucosylase homolog was found. Whether it is involved in the glucosylation of C3-hydroxyl or C28-carboxyl in *I. pubescens* triterpenoids, should be further tested.

To our knowledge, this *de novo* transcriptome assembly described for *I. pubescens* provides the first large scale molecular resource for future genetic studies of this medicinal herb. Comparative transcriptome analysis of two closely related *Ilex* species (*I. pubescens* and *I. asprella*) revealed many interesting orthologs, which might be served as Biobricks for synthetic biology of triterpenoid saponins in the future. It should be noted that only one transcriptome of *I. pubescens, I. asprella*, and *A. paniculata*, respectively, are used in this study. Biological repetition of plant samples and DNA sequence analysis using more characterized genes as objectives will make the results more credible.

## Conclusions

The medicinal plant *I. pubescens* contains a large amount of important triterpenoid saponins. To elucidate the biosynthetic mechanism of these saponins, transcript profiling was obtained. Transcriptome analysis and comparative analysis with a genetically closely related *I. asprella*, eventually with a distant species *A. paniculate*, revealed the promising *OSC, CYP*, and *UGT* candidates involved in the biosynthesis of triterpenoid saponins from *I. pubescens* and *I. asprella*. One new OSC gene from *I. pubescens* was identified as a favoring α-amyrin synthase using the pESC-URA expression system.

Our work provides a rich sequence library of *I. pubescens* and facilitates the studies on biosynthetic mechanism of triterpenoids therein at the transcriptomic level. The putative genes identified in *I. pubescens* will be cloned and characterized in further studies.

## Author contributions

Professor RZ dedicated the identification of origin plant. LW contributed to the tissue samples collection, RNA extraction, data analysis and writing of this manuscript. XY, YX, YC, and JZ offer the help of AS cloning, vector construction, protein expression and GC-MS detection. XZ contributed to establishment of yeast expression system, determination of amyrin by GC-MS and helped to draft the manuscript. HX conceived of the study and prepared the manuscript. WC participated in designing the study and coordination. All authors read and approved the final manuscript.

### Conflict of interest statement

The authors declare that the research was conducted in the absence of any commercial or financial relationships that could be construed as a potential conflict of interest. The reviewer VC and handling Editor declared their shared affiliation, and the handling Editor states that the process nevertheless met the standards of a fair and objective review.

## Supplementary material

Figure S1 **Length distribution of *I. pubescens* transcriptome**.

Figure S2 **Distribution of *E*-value in COG (A)**, KEGG **(B)**, Nr **(C)**, and Swissprot **(D)** databases.

Figure S3 **Numbers of UniGenes annotated by blastx against protein databases (The threshold of *E*-value was set at 10^−5^)**.

Figure S4 **Numbers of UniGenes similar to other species**.

Figure S5 **COG function classification of *I. pubescens* UniGenes**.

Figure S6 **Gene Ontology classification of assembled UniGenes**. Total UniGenes were categorized into three main categories: biological process, cellular component and molecular function.

Figure S7 **Biosynthetic pathways of terpenoids**. MVA pathway, mevalonate pathway; AACT, acetyl-CoA acyltransferase; HMGS, 3-hydroxy-3-methylglutaryl-CoA synthase; HMGR, 3-hydroxy-3-methylglutaryl-CoA reductase; MK, mevalonate kinase; PMK, phosphomevalonate kinase; MDC, mevalonate 5-dinhophate decarboxylase; MEP pathway, 2-Cmethyl-D-erythritol-4-phosphate pathway; G3P, glyceraldehyde-3-phosphate; DXS, 1-deoxy-D-xylulose 5-phosphate synthase; DXR, 1-deoxy-D-xylulose 5-phosphate reductoisomerase; MCT, MEP cytidyltransferase; CMK, 4-(Cytidine 5-diphospho) -2-C-methylerythritol kinase; MDS, 2-C-Methy-D-erythritol 2,4-cyclodiphosphate synthase; HDS, hydroxymethylbutenyl 4-diphosphate synthase; HDR, 4-hydroxy-3 -methylbut-2-enyl diphosphate reductase; IPP, isopentenyl pyropho-sphate; IDI, isopentenyl diphosphate isomerase; DMAPP, dimethylallyl diphosphate; GPPS, geranyl diphosphatesynthase; GPP, geranyldiphosphate; FPPS, farnesyl diphosphate synthase; FPP, diphosphate; SS, squalene synthase; SM, squalene monooxygenase.

Figure S8 **Chemical structures of triterpenoids in *I. pubescens* and *I. asprella***. structure D and E, special in *I. pubescens*; structure A, C, and I, special in *I. asprella;* structure B, F, G, and H, common for *I. pubescens* and *I. asprella*.

Figure S9 **Phylogenetic analysis of the well characterized CYPs involved in pentacyclic triterpenoids biosynthesis**. The statistical methods of this unrooted tree was maximum likehood method, which was tested by bootstrap method and the number of bootstrap replications was 1,000. The oxidation positions on amyrin are indicated in this figure. The open and black stars, open and black triangles indicate the cytochrome P450 clustered together with the CYP85, CYP71, CYP51, and CYP72 clans, respectively. Aa, *Artemisia annua*; Ah, *Arachis hypogaea*; Al, *Arabidopsis lyrata* subsp. *lyrata*; As, *Avena strigose*; At, *Arabidopsis thaliana*; Bf, *Bupleurum falcatum*; Ca, *Cicer arietinum*; Cr, *Catharanthus roseus*; Gg, *Glycyrrhiza glabra*; Gm, *Glycine max*; Gu, *Glycyrrhiza uralensis*; Lc, *Lens culinaris*; Ml, *Maesa lanceolate*; Mt, *Medicago truncatula*; Pg, *Panax ginseng*; Ps, *Pisum sativum*; Pv, *Phaseolus vulgaris*; Vv, *Vitis vinifera*.

Figure S10 **Characteristics of CYPs in *I. pubescens*. (A)** Distribution of the CYP families; **(B)** Length distribution of these CYPs; **(C)** RPKM distribution of the CYPs; **(D)** GO classify of CYPs in *I. pubescens*.

Figure S11 **Phylogenetic analysis of the well-characterized UGTs involved in pentacyclic triterpenoids biosynthesis**. The statistical methods of this unrooted tree were the same as Figure S9 and glucosylation positions on amyrin are indicated. The open stars, black stars and black triangles indicate the UGTs clustered together with the UGT73, UGT74, and UGT91 clans, respectively. Bv, *Barbarea vulgaris* subsp. *arcuate*; Gg, *Glycyrrhiza glabra*; Gm, *Glycine max*; Mt, *Medicago truncatula*; Sv, *Saponaria vaccaria*.

Figure S12 **Distribution of homologous genes and specific genes in *I. pubescens* and *I. asprella***.

Figure S13 **Distribution of Ka/Ks ratio**. Ortholog pairs with Ka/Ks ratio >1 are above the full line, while ortholog pairs with Ka/Ks ratio between 0.5 and 1 reside between the full and imaginary lines.

Figure S14 **Phylogenetic analysis of the well-characterized CYPs involved in diterpenoids and triterpenoids biosynthesis**. The statistical methods of this unrooted tree was the same as Figure S9. The black stars indicate the CYPs were involved in diterpenoids biosynthesis. Gb, *Ginkgo bilobaSm*; Sm, *Salvia miltiorrhiza*; Tca, *Taxus canadensis*; Tcu, *Taxuscuspidata*; Tx, *Taxusx media*.

Figure S15 **Comparison of amino acid sequence of *IpAS*1 amd *IaAS*1**. QW, DCTAE, MWCYCR and PVRXXE motif were demonstrated with red solid frame, red line, black solid line and black line, respectively.

Table S1 **Identified pentacyclic triterpenoids in *I. pubescens***.

Table S2 **Identified pentacyclic triterpenoids in *I. asprella***.

Table S3 **Information of Characterized CYPs and UGTs for Blastp analysis**.

Table S4 **Designed primers for IpAS1 cloning**.

Table S5 **The CYPs with length >1,000 bp and classified as CYP72 clan and CYP85 clan in *I. pubescens***.

Table S6 **Putative Ilex-genus-specific *CYP*s**.
