# Supplementary figures and images for: Transcriptomic Comparison Reveals Candidate Genes for Triterpenoid Biosynthesis in Two Closely Related Ilex Species
Source: Front Plant Sci. 2017 Apr 28;8:634. doi: 10.3389/fpls.2017.00634 (PMC5408325; doi:10.3389/fpls.2017.00634)

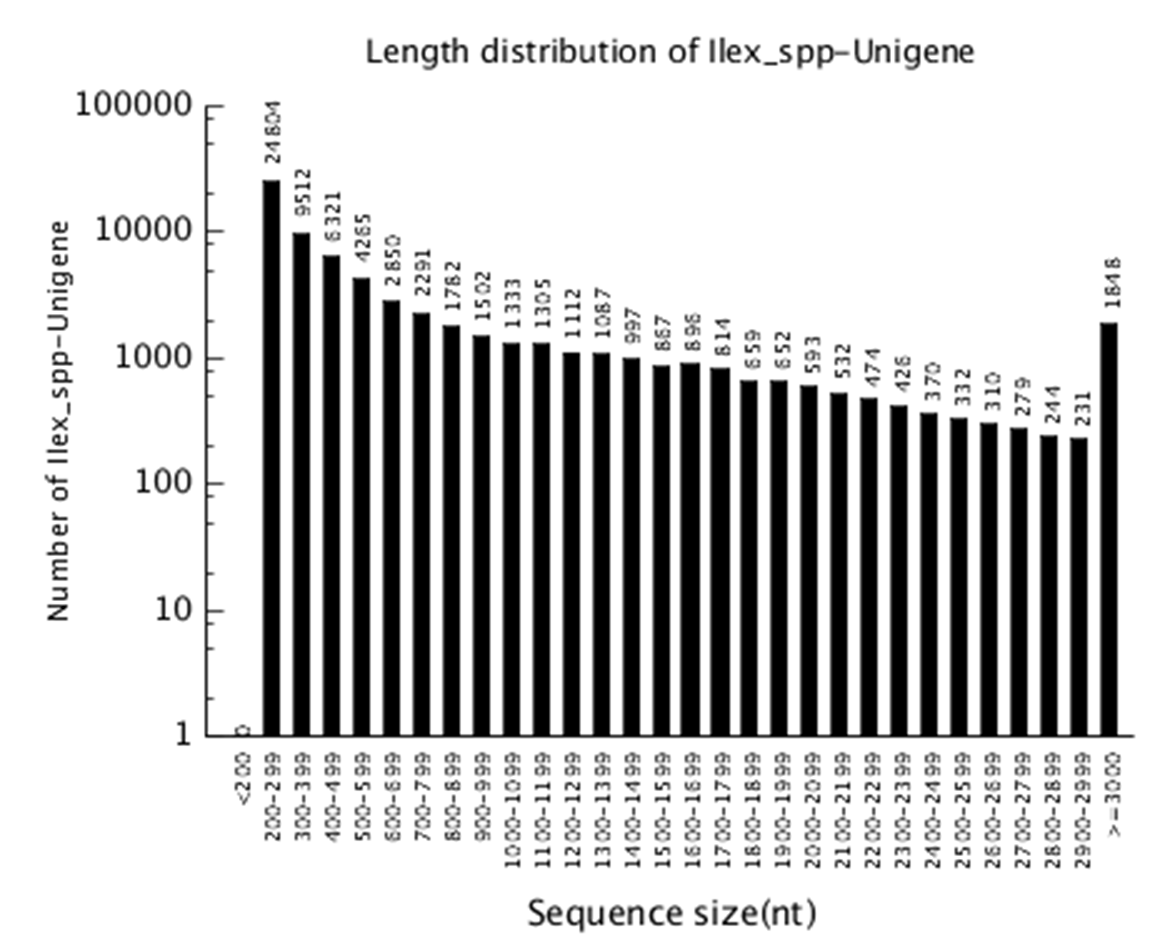

Supplement: Figure S1 — Length distribution of I. pubescens transcriptome. [file Image1.TIF]

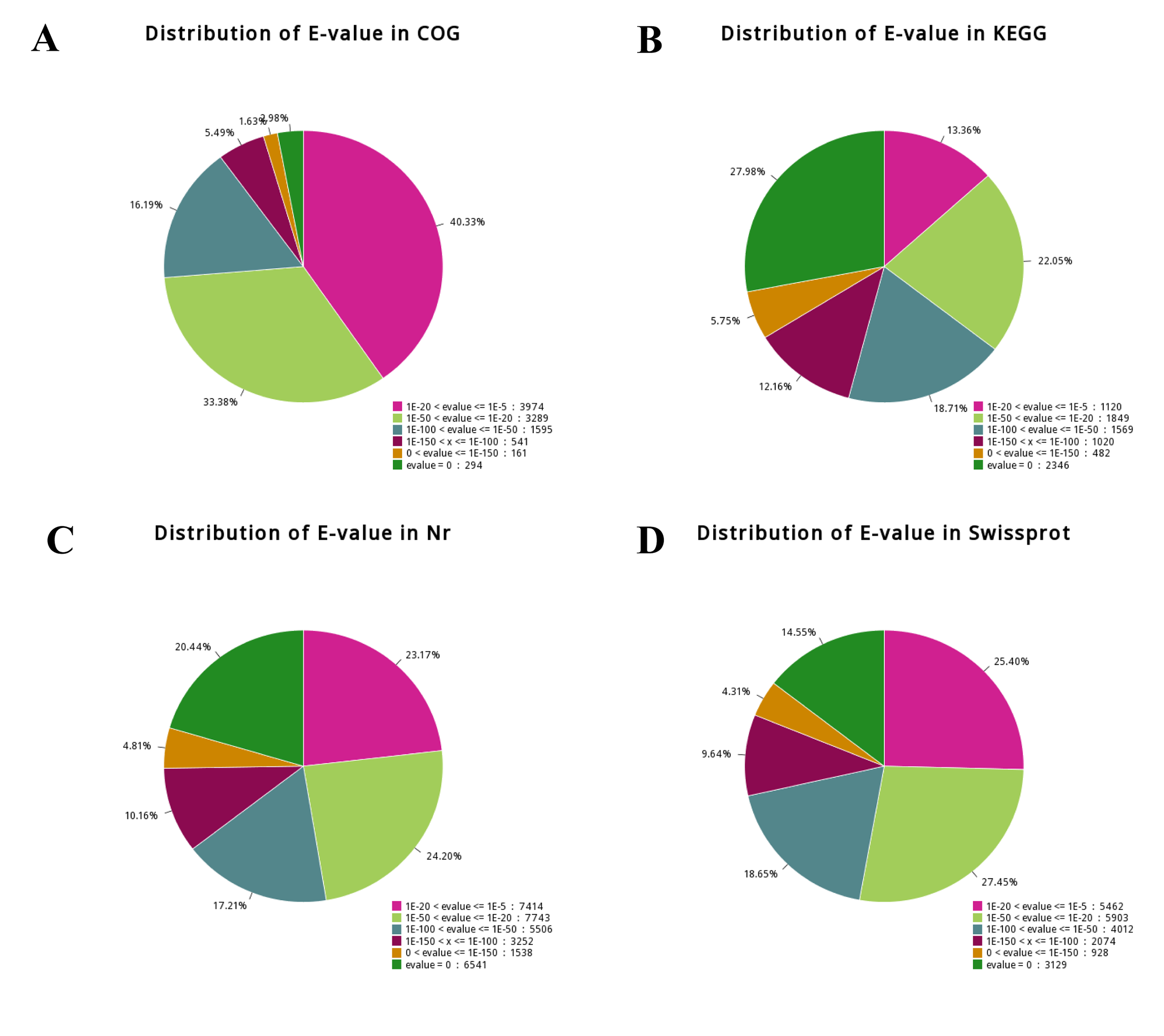

Supplement: Figure S2 — Distribution of E-value in COG (A), KEGG (B), Nr (C), and Swissprot (D) databases. [file Image2.TIF]

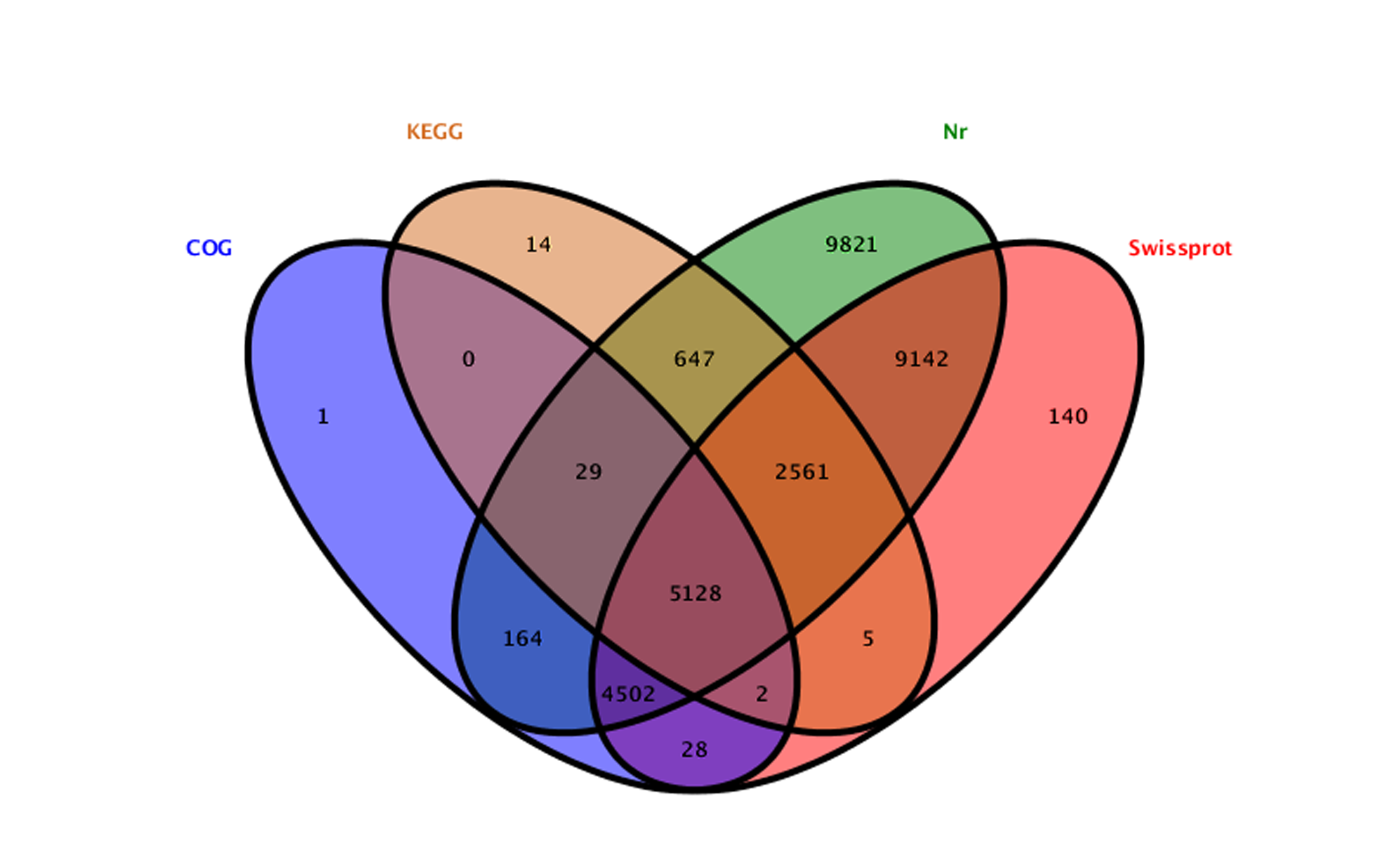

Supplement: Figure S3 — Numbers of UniGenes annotated by blastx against protein databases (The threshold of E-value was set at 10−5). [file Image3.TIFF]

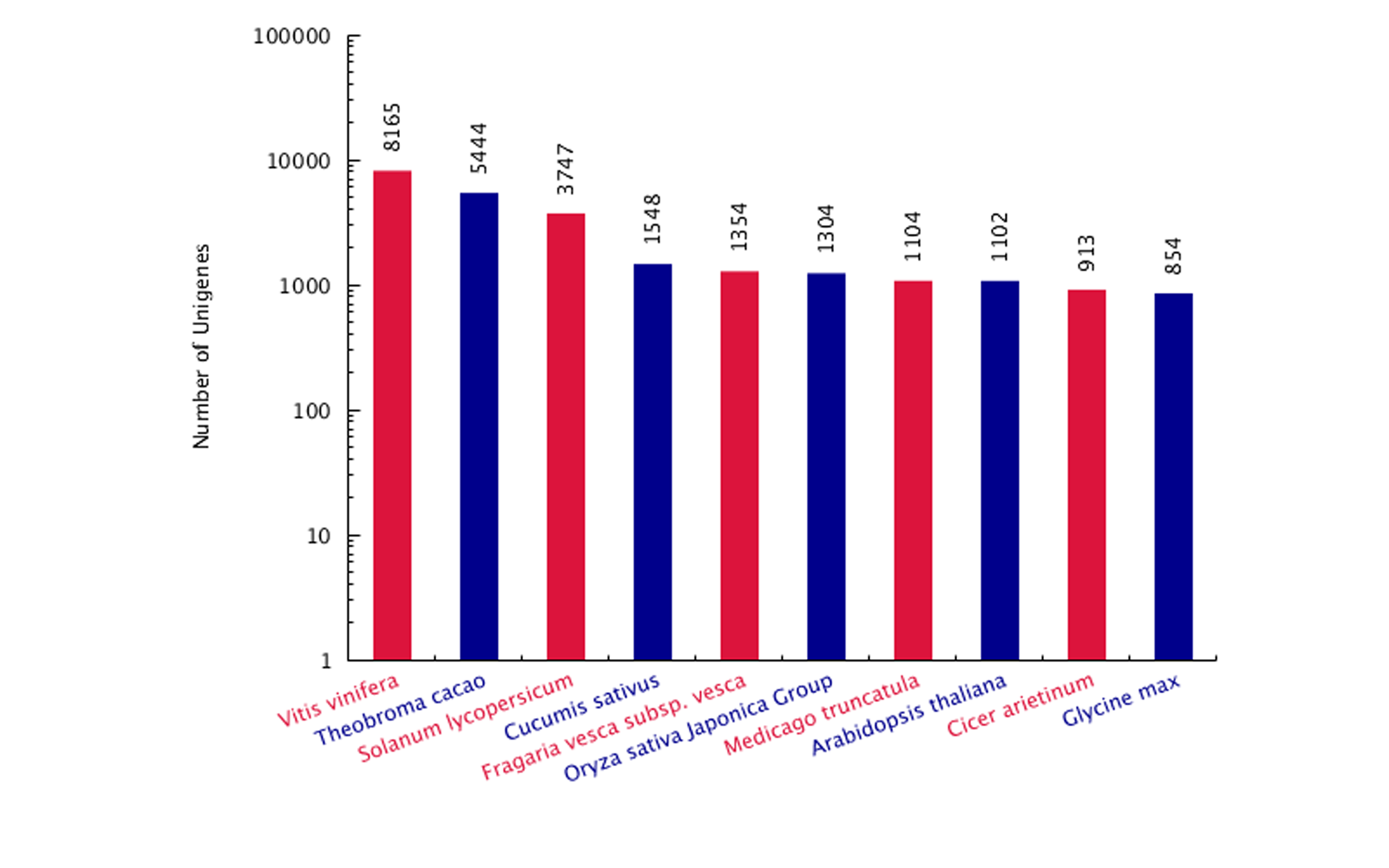

Supplement: Figure S4 — Numbers of UniGenes similar to other species. [file Image4.TIFF]

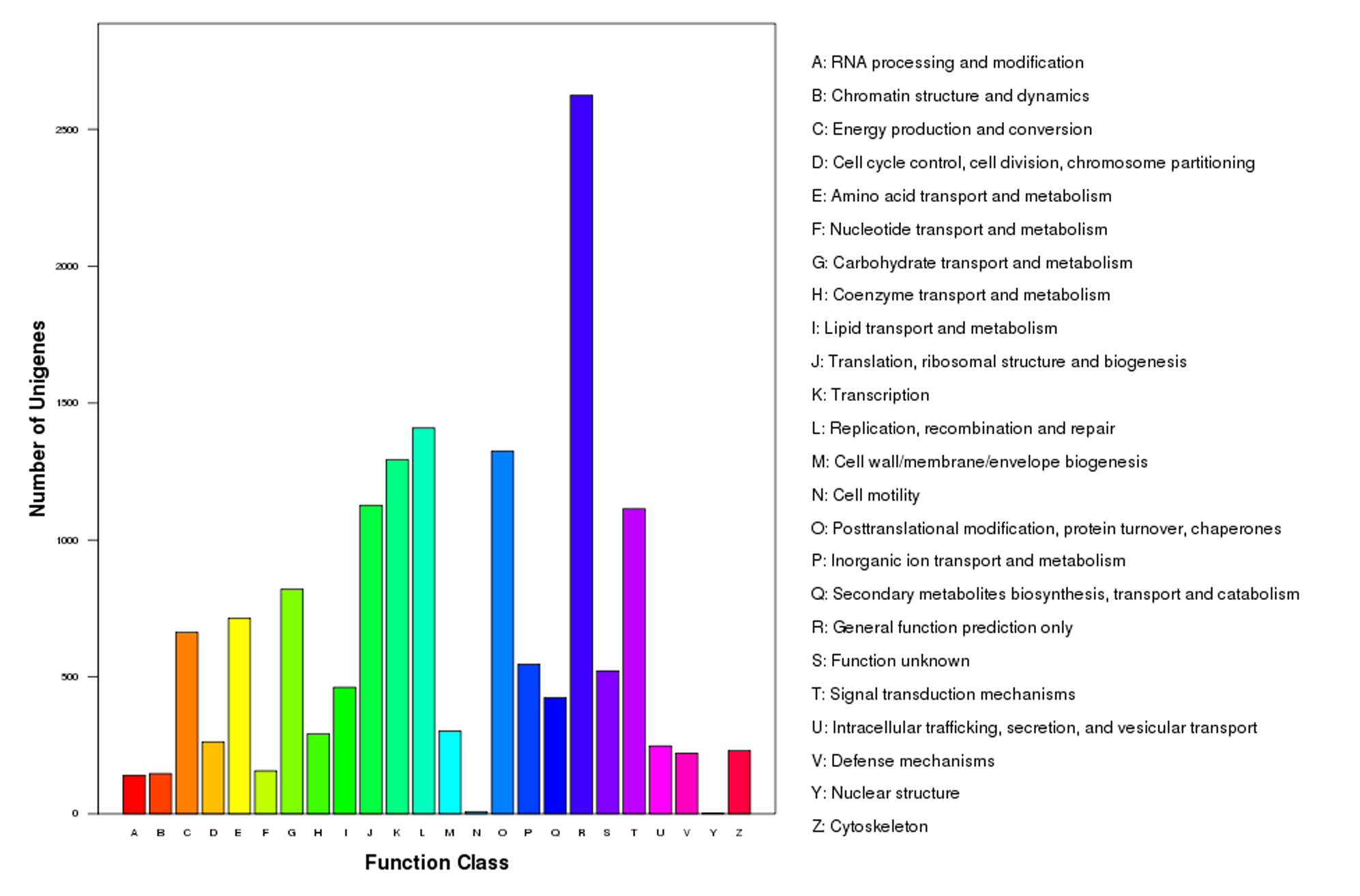

Supplement: Figure S5 — COG function classification of I. pubescens UniGenes. [file Image5.TIFF]

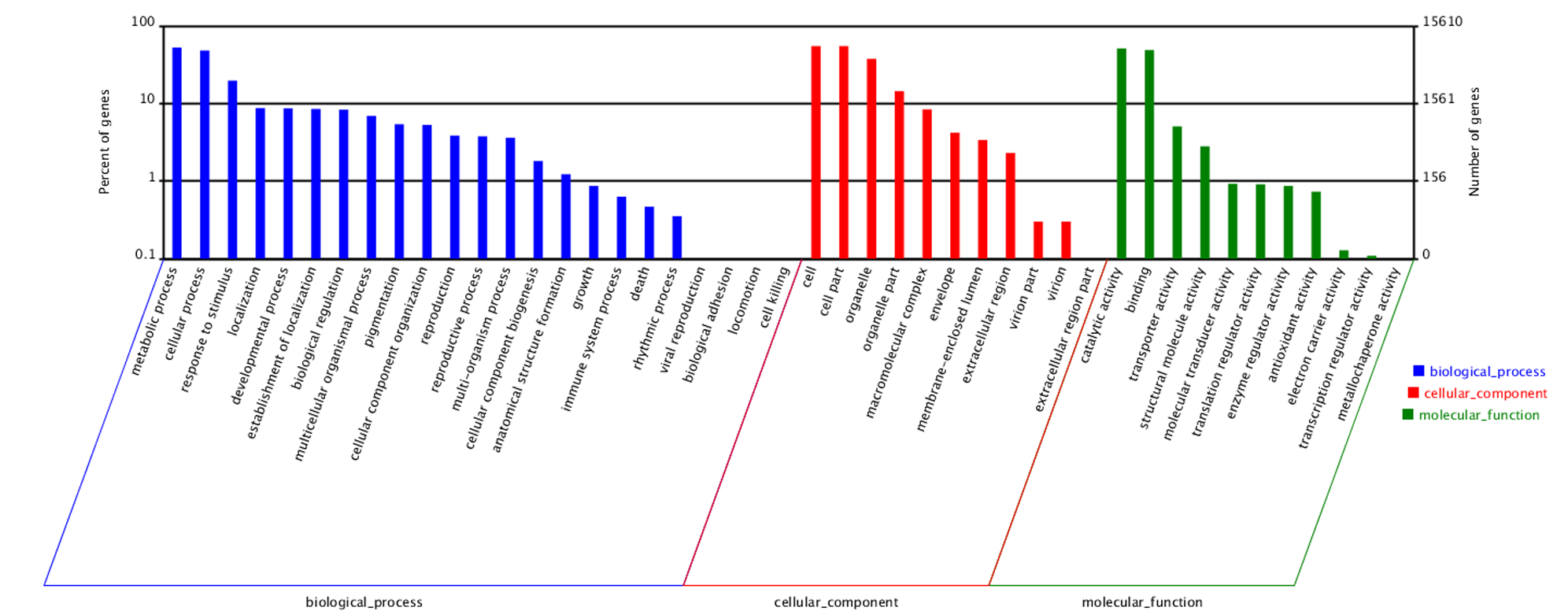

Supplement: Figure S6 — Gene Ontology classification of assembled UniGenes. Total UniGenes were categorized into three main categories: biological process, cellular component and molecular function. [file Image6.TIFF]

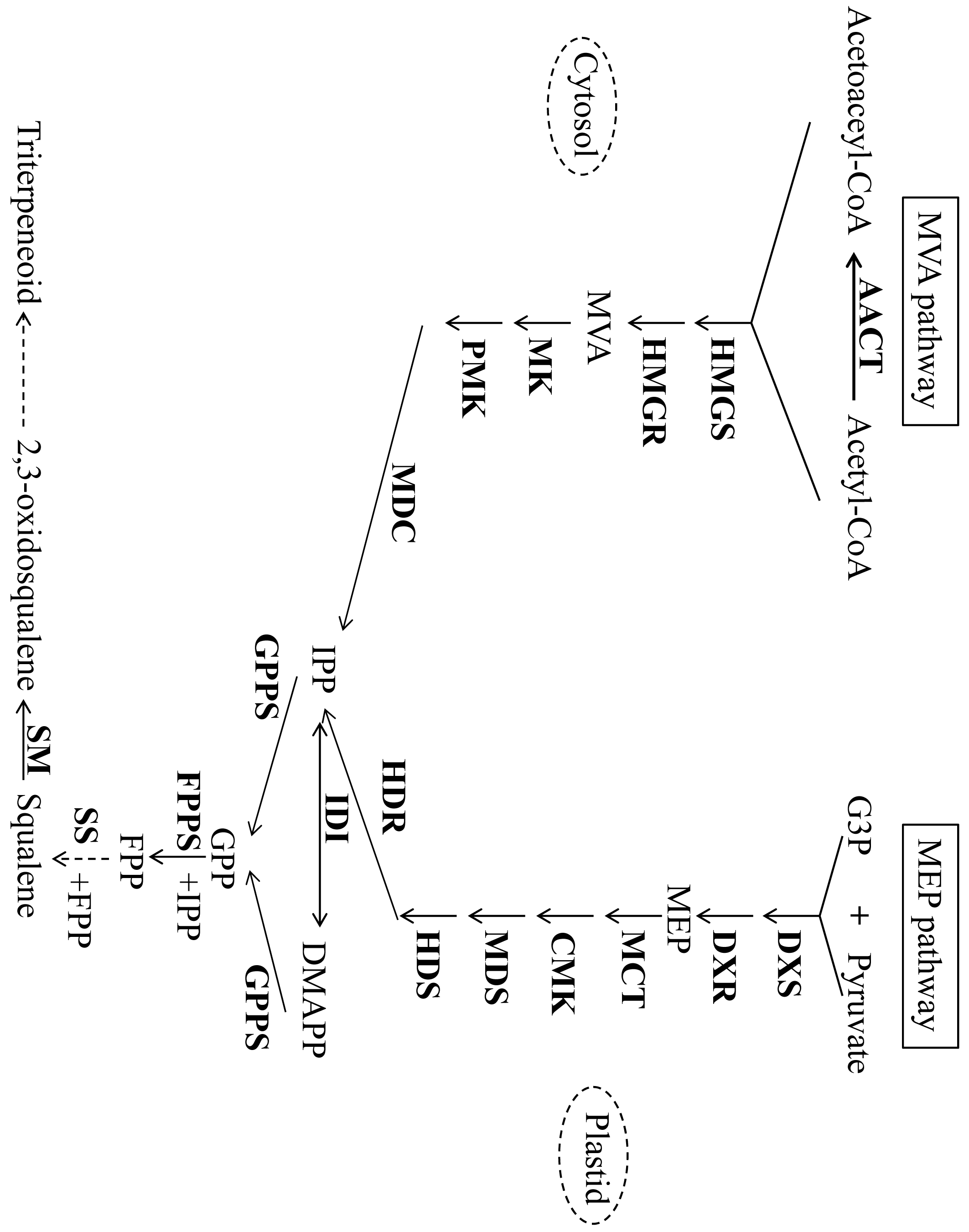

Supplement: Figure S7 — Biosynthetic pathways of terpenoids. MVA pathway, mevalonate pathway; AACT, acetyl-CoA acyltransferase; HMGS, 3-hydroxy-3-methylglutaryl-CoA synthase; HMGR, 3-hydroxy-3-methylglutaryl-CoA reductase; MK, mevalonate kinase; PMK, phosphomevalonate kinase; MDC, mevalonate 5-dinhophate decarboxylase; MEP pathway, 2-Cmethyl-D-erythritol-4-phosphate pathway; G3P, glyceraldehyde-3-phosphate; DXS, 1-deoxy-D-xylulose 5-phosphate synthase; DXR, 1-deoxy-D-xylulose 5-phosphate reductoisomerase; MCT, MEP cytidyltransferase; CMK, 4-(Cytidine 5-diphospho) -2-C-methylerythritol kinase; MDS, 2-C-Methy-D-erythritol 2,4-cyclodiphosphate synthase; HDS, hydroxymethylbutenyl 4-diphosphate synthase; HDR, 4-hydroxy-3 -methylbut-2-enyl diphosphate reductase; IPP, isopentenyl pyropho-sphate; IDI, isopentenyl diphosphate isomerase; DMAPP, dimethylallyl diphosphate; GPPS, geranyl diphosphatesynthase; GPP, geranyldiphosphate; FPPS, farnesyl diphosphate synthase; FPP, diphosphate; SS, squalene synthase; SM, squalene monooxygenase. [file Image7.TIF]

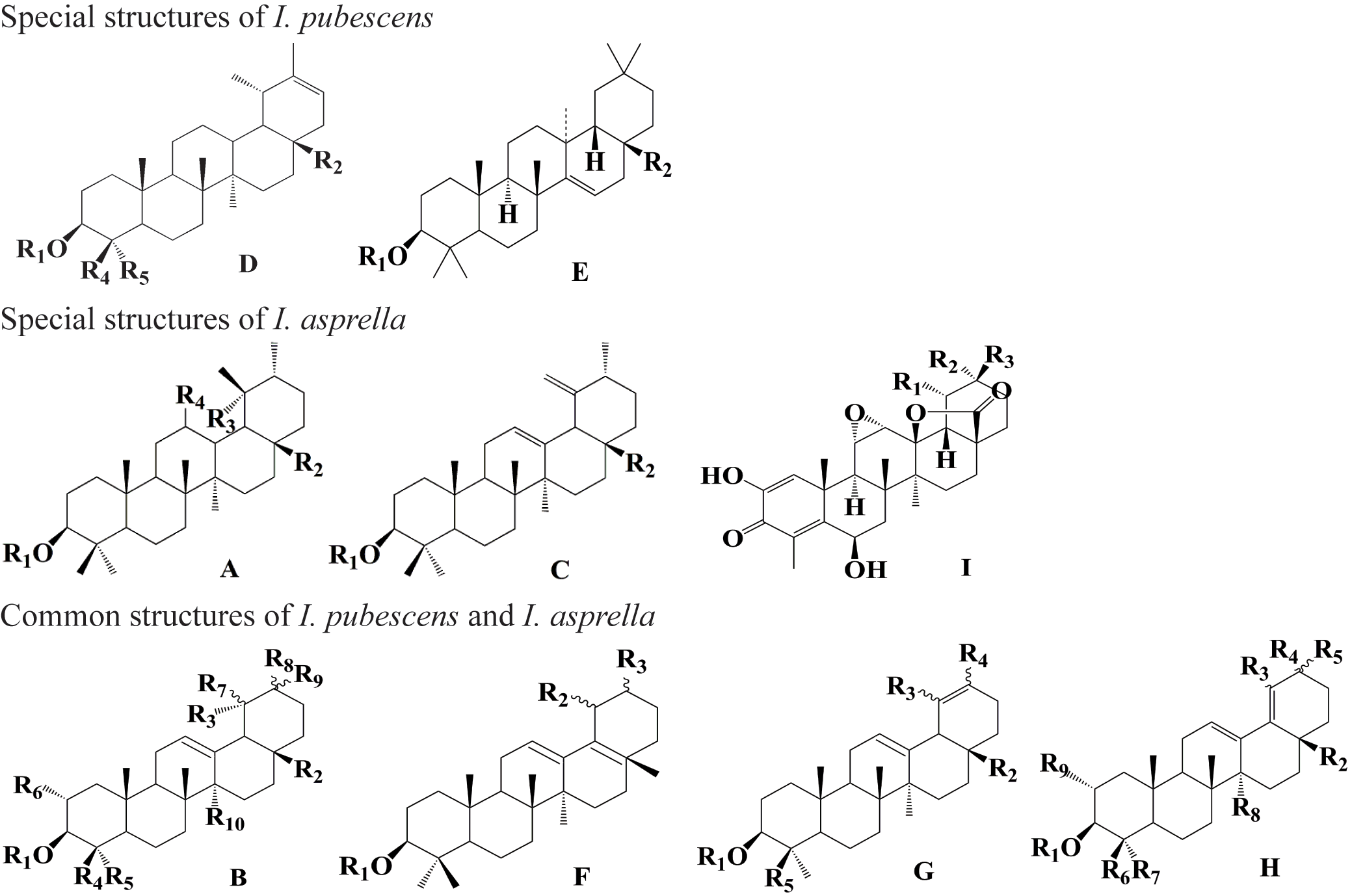

Supplement: Figure S8 — Chemical structures of triterpenoids in I. pubescens and I. asprella. structure D and E, special in I. pubescens; structure A, C, and I, special in I. asprella; structure B, F, G, and H, common for I. pubescens and I. asprella. [file Image8.TIF]

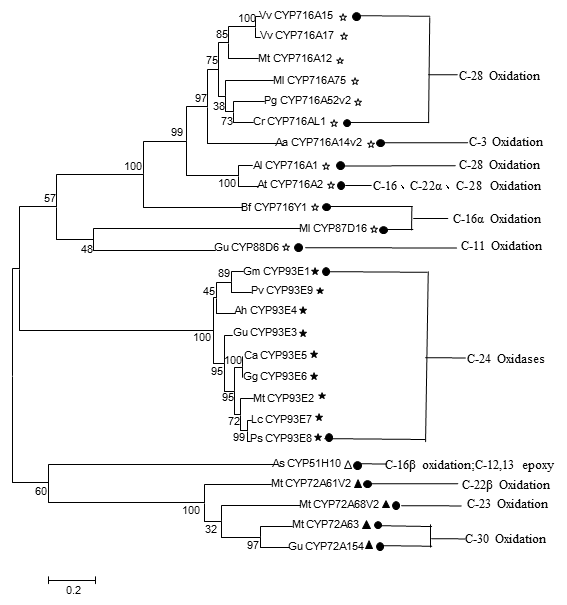

Supplement: Figure S9 — Phylogenetic analysis of the well characterized CYPs involved in pentacyclic triterpenoids biosynthesis. The statistical methods of this unrooted tree was maximum likehood method, which was tested by bootstrap method and the number of bootstrap replications was 1,000. The oxidation positions on amyrin are indicated in this figure. The open and black stars, open and black triangles indicate the cytochrome P450 clustered together with the CYP85, CYP71, CYP51, and CYP72 clans, respectively. Aa, Artemisia annua; Ah, Arachis hypogaea; Al, Arabidopsis lyrata subsp. lyrata; As, Avena strigose; At, Arabidopsis thaliana; Bf, Bupleurum falcatum; Ca, Cicer arietinum; Cr, Catharanthus roseus; Gg, Glycyrrhiza glabra; Gm, Glycine max; Gu, Glycyrrhiza uralensis; Lc, Lens culinaris; Ml, Maesa lanceolate; Mt, Medicago truncatula; Pg, Panax ginseng; Ps, Pisum sativum; Pv, Phaseolus vulgaris; Vv, Vitis vinifera. [file Image9.TIF]

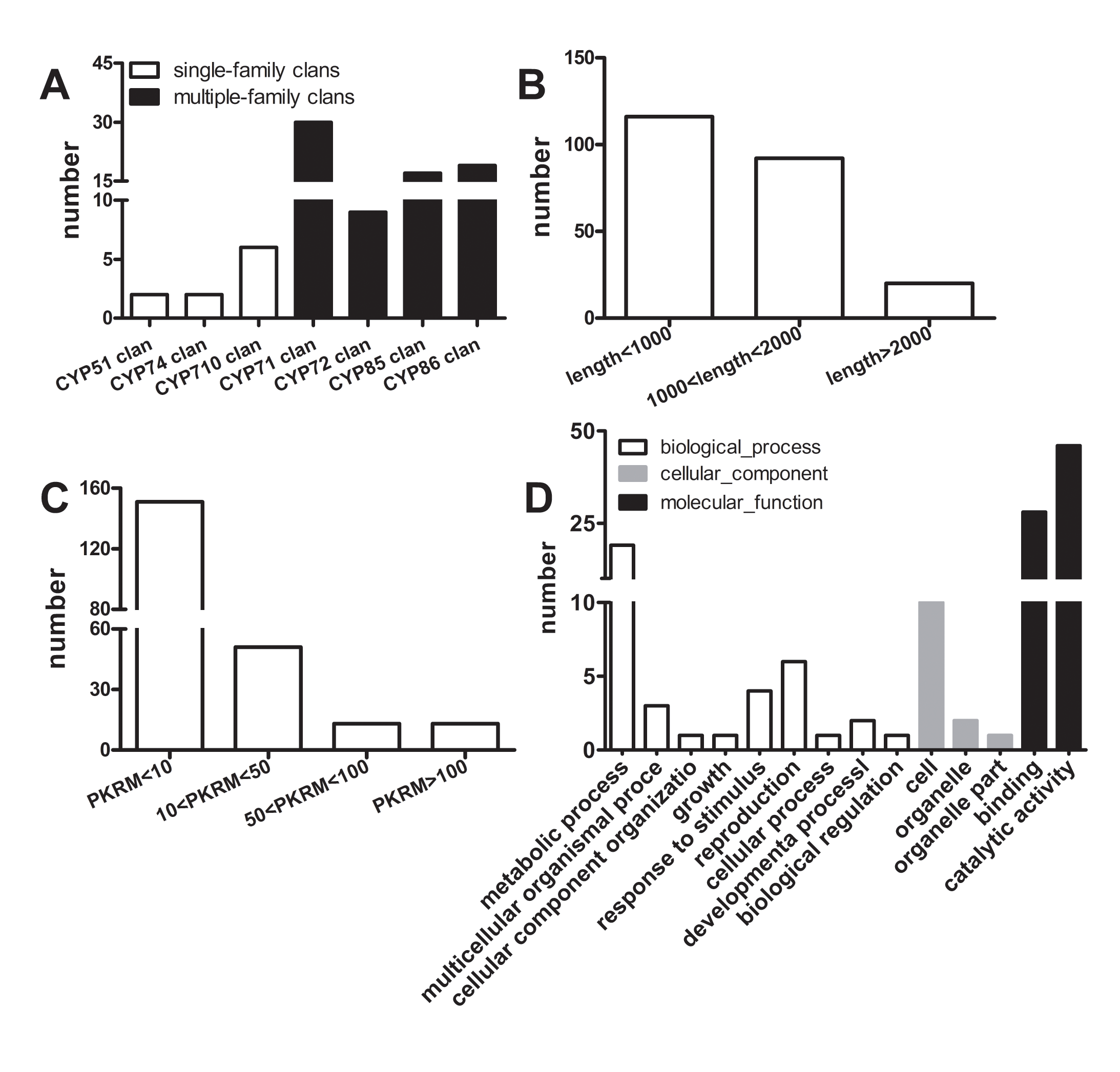

Supplement: Figure S10 — Characteristics of CYPs in I. pubescens. (A) Distribution of the CYP families; (B) Length distribution of these CYPs; (C) RPKM distribution of the CYPs; (D) GO classify of CYPs in I. pubescens. [file Image10.TIF]

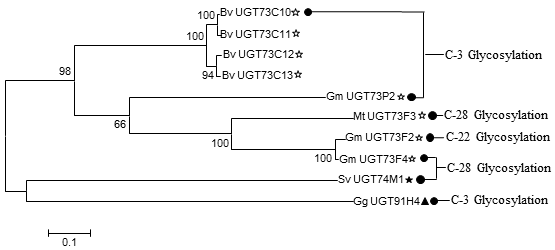

Supplement: Figure S11 — Phylogenetic analysis of the well-characterized UGTs involved in pentacyclic triterpenoids biosynthesis. The statistical methods of this unrooted tree were the same as Figure S9 and glucosylation positions on amyrin are indicated. The open stars, black stars and black triangles indicate the UGTs clustered together with the UGT73, UGT74, and UGT91 clans, respectively. Bv, Barbarea vulgaris subsp. arcuate; Gg, Glycyrrhiza glabra; Gm, Glycine max; Mt, Medicago truncatula; Sv, Saponaria vaccaria. [file Image11.TIF]

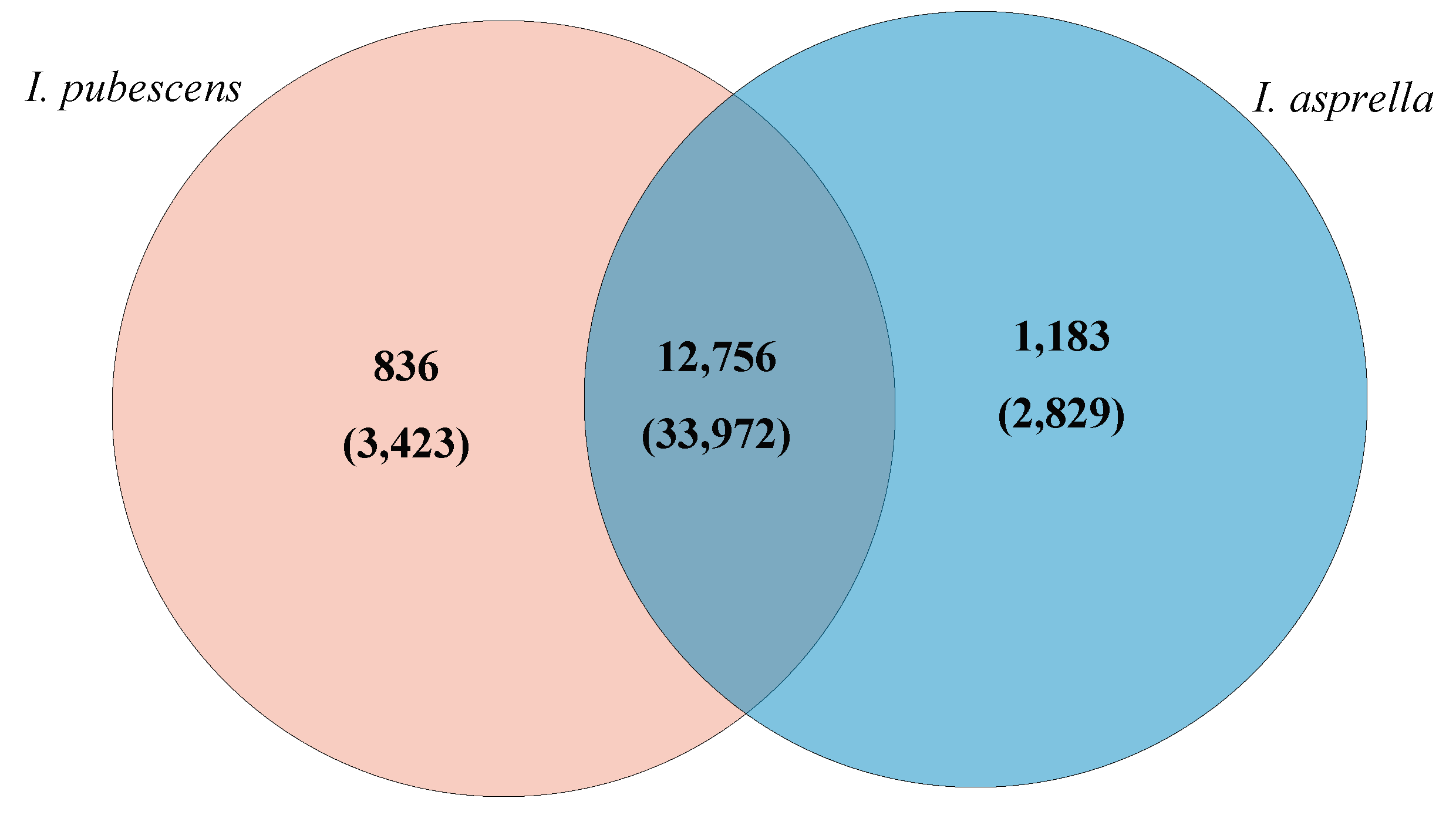

Supplement: Figure S12 — Distribution of homologous genes and specific genes in I. pubescens and I. asprella. [file Image12.TIF]

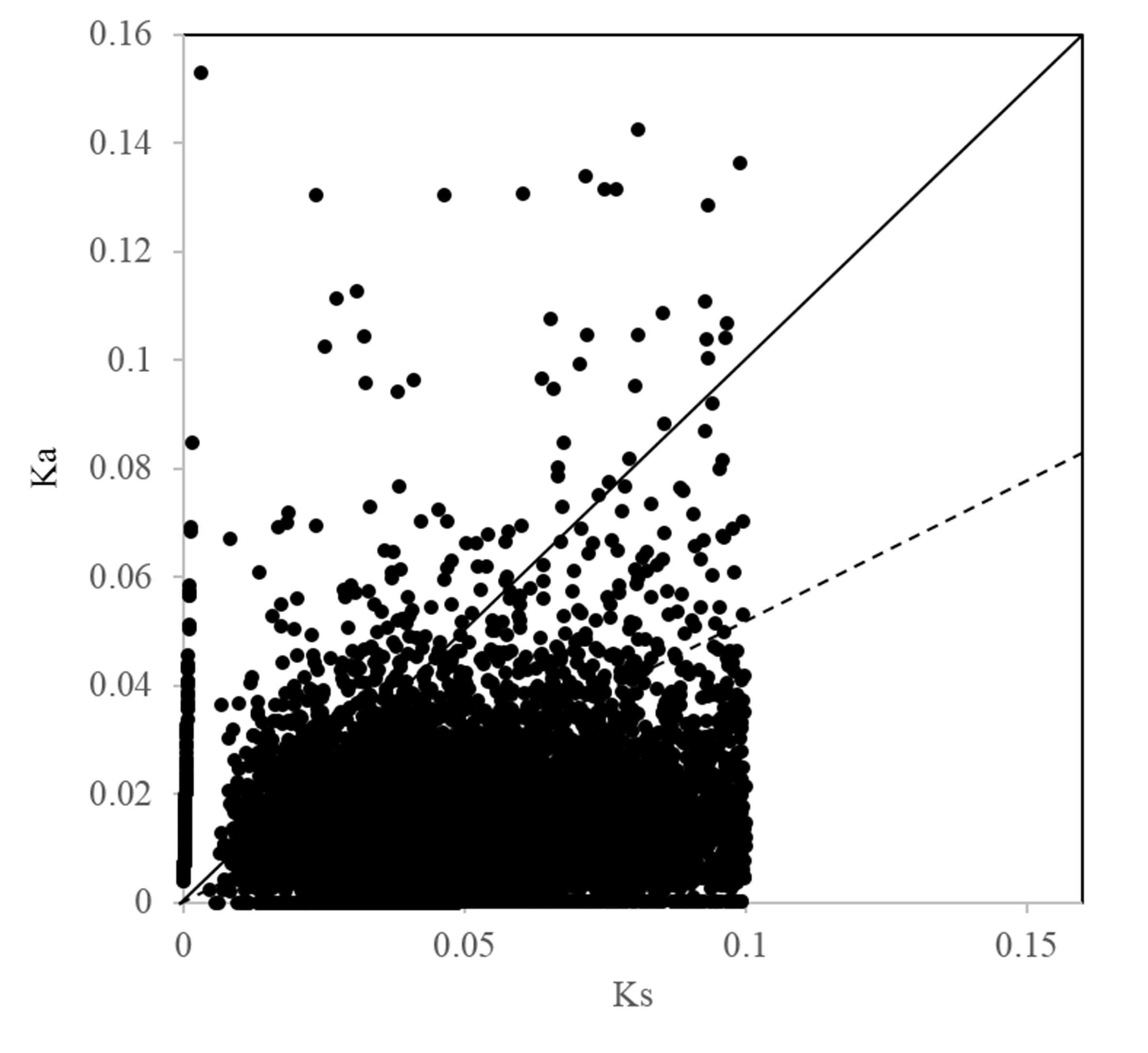

Supplement: Figure S13 — Distribution of Ka/Ks ratio. Ortholog pairs with Ka/Ks ratio >1 are above the full line, while ortholog pairs with Ka/Ks ratio between 0.5 and 1 reside between the full and imaginary lines. [file Image13.TIF]

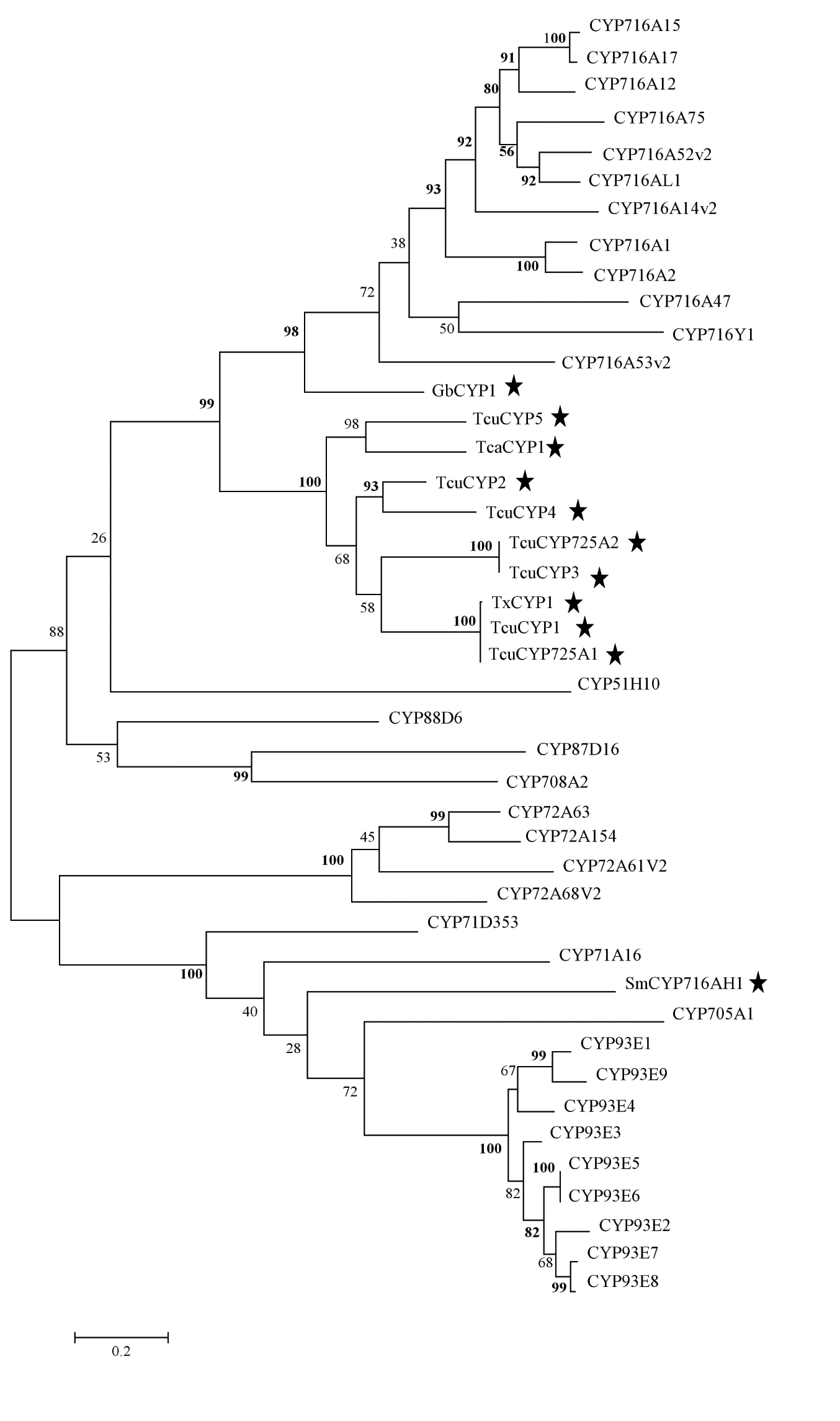

Supplement: Figure S14 — Phylogenetic analysis of the well-characterized CYPs involved in diterpenoids and triterpenoids biosynthesis. The statistical methods of this unrooted tree was the same as Figure S9. The black stars indicate the CYPs were involved in diterpenoids biosynthesis. Gb, Ginkgo bilobaSm; Sm, Salvia miltiorrhiza; Tca, Taxus canadensis; Tcu, Taxuscuspidata; Tx, Taxusx media. [file Image14.TIF]

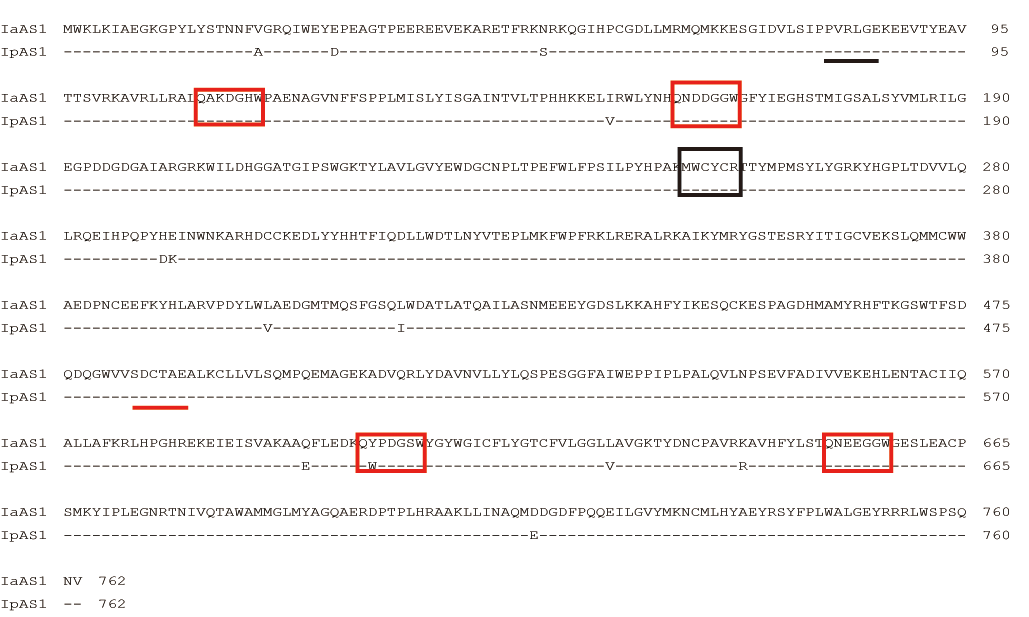

Supplement: Figure S15 — Comparison of amino acid sequence of IpAS1 amd IaAS1. QW, DCTAE, MWCYCR and PVRXXE motif were demonstrated with red solid frame, red line, black solid line and black line, respectively. [file Image15.TIF]
