# Supplementary material for: Transcriptomic Comparison Reveals Candidate Genes for Triterpenoid Biosynthesis in Two Closely Related Ilex Species
Source: Front Plant Sci. 2017 Apr 28;8:634. doi: 10.3389/fpls.2017.00634 (PMC5408325; doi:10.3389/fpls.2017.00634)
Supplement: Table S1 — Identified pentacyclic triterpenoids in I. pubescens. [file Table1.DOC]

**Table S1. Identified pentacyclic triterpenoids** **in** ***I. pubescens***.

| type | Name | R_1_ | R_2_ | R_3_ | R_4_ | R_5_ | R_6_ | R_7_ | R_8_ | R_9_ | R_10_ |
| --- | --- | --- | --- | --- | --- | --- | --- | --- | --- | --- | --- |
| B | Ursolic acid（Arthur *et al.*, 1956） | H | COOH | H | CH_3_ | CH_3_ | H | β-CH_3_ | α-CH_3_ | β-H | CH_3_ |
|  | Oleanolic acid（Arthur *et al*., 1956） | H | COOH | H | CH_3_ | CH_3_ | H | β-H | β-CH_3_ | α-CH_3_ | CH_3_ |
|  | Ilexolic acid B(Feng, 2012) | H | COOH | H | COOH | CH_3_ | H | β-OH | β-CH_3_ | α-CH_3_ | CH_3_ |
|  | Chaenomelogenin A(Feng, 2012) | H | COOH | H | CH_3_ | COOH | OH | β-H | β-CH_3_ | α-CH_3_ | CH_3_ |
|  | 23-Aldehydepolomic acid(Feng, 2012) | H | COOH | OH | CHO | CH_3_ | H | β-CH_3_ | α-CH_3_ | β-H | CH_3_ |
|  | Ilexgenin A(Hidaka *et al*., 1986) | H | COOH | OH | CH_3_ | COOH | H | β-CH_3_ | α-CH_3_ | β-H | CH_3_ |
|  | Siaresinolic acid-28-O-β-D-glucopyranosyl ester(Wu *et al*., 2015) | H | COOGlc | OH | CH_3_ | CH_3_ | H | β-H | β-CH_3_ | α-CH_3_ | CH_3_ |
|  | Ilexgenin B 28-O-β-D-glucopyranosyl ester(Zhao *et al*., 2011) | H | COOGlc | OH | CH_3_ | CH_3_ | H | β-CH_3_ | β-CH_3_ | α-H | CH_3_ |
|  | Ilexoside E(Han *et al*., 1987) | H | COOGlc | OH | COOH | CH_3_ | H | β-CH_3_ | α-CH_3_ | β-H | CH_3_ |
|  | 3β,19α-dihydroxyolean-12-ene-24,28-dioic-28-O-β-D-glucopyranoside(Zhou *et al*., 2012) | H | COOGlc | OH | COOH | CH_3_ | H | α-H | β-CH_3_ | α-CH_3_ | CH_3_ |
|  | Pedunculoside(Cai *et al*., 2001) | H | COOGlc | OH | CH_3_ | CH_2_OH | H | β-CH_3_ | β-CH_3_ | α-H | CH_3_ |
|  | Ilexsaponin A_1_(Hidaka *et al*., 1986) | H | COOGlc | OH | CH_3_ | COOH | H | β-CH_3_ | α-CH_3_ | β-H | CH_3_ |
|  | Chaenomeloside A(Feng, 2012) | H | COOGlc | H | CH_3_ | COOH | OH | β-H | β-CH_3_ | α-CH_3_ | CH_3_ |
|  | 6’-O-acetyl-ilexsaponin A_1_(Jiang *et al*., 2008) | H | COOGlc-6’-O-acetyl | OH | COOH | CH_3_ | H | β-CH_3_ | α-CH_3_ | β-H | CH_3_ |
|  | Ilexpublesnin B(Zhang *et al*., 2010) | H | COOXyl-Glc | OH | CHO | CH_3_ | H | β-CH_3_ | α-CH_3_ | β-H | CH_3_ |
|  | Cauloside A(Lin *et al*., 2015) | Ara | COOH | H | CH_3_ | CH_2_OH | H | β-H | α-CH_3_ | β-CH_3_ | CH_3_ |
|  | Ziyu-glycoside I(Zhang *et al*., 2011) | Ara | COOGlc | OH | CH_3_ | CH_3_ | H | β-CH_3_ | α-CH_3_ | β-H | CH_3_ |
|  | Ilexpublesnin R(Zhou *et al*., 2014) | sulfo-Ara | COOGlc | OH | CH_3_ | CH_3_ | H | β-H | α-CH_3_ | β-CH_3_ | CH_3_ |
|  | Ilexpublesnin H(Zhou *et al*., 2013) | sulfo-Ara | COOGlc | OH | CH_3_ | CH_3_ | H | β-CH_3_ | β-CH_3_ | α-H | CH_3_ |
|  | Ilexpublesnin Q(Zhou *et al*., 2014) | sulfo-Ara | COOGlc | OH | CH_3_ | CH_3_ | H | β-CH_3_ | α-CH_3_ | β-H | CH_3_ |
|  | Calenduloside E (Zhao *et al*., 2011) | GlcA | COOH | H | CH_3_ | CH_3_ | H | β-H | β-CH_3_ | α-CH_3_ | CH_3_ |
|  | Ilexpublesnin D(Zhou *et al*., 2013) | GlcA | COOH | OH | CHO | CH_3_ | H | β-CH_3_ | α-CH_3_ | β-H | CH_3_ |
|  | Ilexpublesnins M(Zhou *et al*., 2013) | GlcA | COOH | OH | CHO | CH_3_ | H | β-H | β-CH_3_ | α-CH_3_ | CH_3_ |
|  | Chikusetsusaponin IVa(Wang *et al*., 2008) | GlcA | COOGlc | H | CH_3_ | CH_3_ | H | β-H | β-CH_3_ | α-CH_3_ | CH_3_ |
| type | Name | R_1_ | R_2_ | R_3_ | R_4_ | R_5_ | R_6_ | R_7_ | R_8_ | R_9_ | R_10_ |
| B | Ilexpublesnin C(Zhou *et al*., 2013) | GlcA | COOGlc | OH | CHO | CH_3_ | H | β-CH_3_ | α-CH_3_ | β-H | CH_3_ |
|  | Ilexpublesnin L(Zhou *et al*., 2013) | GlcA | COOGlc | OH | CHO | CH_3_ | H | β-H | β-CH_3_ | α-CH_3_ | CH_3_ |
|  | Chikusetsusaponin IVa methyl ester(Wu *et al*., 2015) | GlcA-6’-COOCH_3_ | COOGlc | H | CH_3_ | CH_3_ | H | β-H | β-CH_3_ | α-CH_3_ | CH_3_ |
|  | Ilexsaponin A_2_(Zhou *et al*., 2013b) | Glc | COOH | OH | COOH | CH_3_ | H | β-CH_3_ | α-CH_3_ | β-H | CH_3_ |
|  | Lucyoside H(Zhang *et al*., 2011) | Glc | COOGlc | H | CH_3_ | CH_3_ | H | β-H | β-CH_3_ | α-CH_3_ | CH_3_ |
|  | Mussaendoside R(Zhang *et al*., 2011) | Glc | COOGlc | OH | CH_3_ | CH_3_ | H | β-CH_3_ | β-CH_3_ | α-H | CH_3_ |
|  | Ilexoside B(Lin *et al*., 2015) | Xyl | COOH | OH | CH_3_ | CH_3_ | H | β-CH_3_ | α-CH_3_ | β-H | CH_3_ |
|  | Ilexgenin B-3-O-β-D-Xylpranoside(Feng *et al*., 2008) | Xyl | COOH | OH | CH_3_ | CH_3_ | H | β-CH_3_ | β-CH_3_ | α-H | CH_3_ |
|  | Ilexpublesnin P(Zhou *et al*., 2014) | Xyl | COOH | OH | CH_2_OH | CH_3_ | H | β-CH_3_ | β-CH_3_ | α-H | CH_3_ |
|  | Ilexpublesnin D(Zhao *et al*., 2012) | Xyl | COOH | OH | CH_2_OH | CH_3_ | H | β-H | β-CH_3_ | α-CH_3_ | CH_3_ |
|  | Ilexpublesnin S(Wu *et al*., 2015) | Xyl | COOH | OH | CHO | CH_3_ | H | β-CH_3_ | β-CH_3_ | α-H | CH_3_ |
|  | Ilexoside A(Han *et al*., 1987) | Xyl | COOGlc | OH | CH_3_ | CH_3_ | H | β-CH_3_ | β-CH_3_ | α-H | CH_3_ |
|  | Ilexsaponin C(Feng *et al*., 2008b) | Xyl | COOGlc | OH | CH_3_ | CH_2_OH | H | β-H | β-CH_3_ | α-CH_3_ | CH_3_ |
|  | Ilexpublesnin A(Zhang *et al*., 2010) | Xyl | COOGlc | OH | CHO | CH_3_ | H | β-CH_3_ | α-CH_3_ | β-H | CH_3_ |
|  | Ilexpublesnin G(Zhou *et al*., 2013) | Xyl | COOGlc | OH | CH_2_OH | CH_3_ | H | β-CH_3_ | β-CH_3_ | α-H | CH_3_ |
|  | Ilexpublesnin I(Zhou *et al*., 2013) | sulfo-Xyl | COOGlc | OH | CH_3_ | CH_3_ | H | β-CH_3_ | β-CH_3_ | α-H | CH_3_ |
|  | Ilexside I(Lin *et al*., 2015) | Glc-Ara | COOH | OH | CH_3_ | CH_3_ | H | β-CH_3_ | α-CH_3_ | β-H | CH_3_ |
|  | Flaccidoside I(Lin *et al*., 2015) | Glc-Xyl | COOH | H | CH_3_ | CH_3_ | H | β-H | β-CH_3_ | α-CH_3_ | CH_3_ |
|  | Ilexoside D(Han *et al*., 1987) | Glc-Xyl | COOH | OH | CH_3_ | CH_3_ | H | β-CH_3_ | β-CH_3_ | α-H | CH_3_ |
|  | Ilexoside K(Han *et al*., 1987) | Glc-Xyl | COOGlc | OH | CH_3_ | CH_3_ | H | β-CH_3_ | β-CH_3_ | α-H | CH_3_ |
|  | Ilexoside J(Han *et al*., 1987) | Rha-Glc | COOH | OH | CH_3_ | CH_3_ | H | β-CH_3_ | β-CH_3_ | α-H | CH_3_ |
|  | Ilexsaponin B_1_( Hidaka *et al*., 1987) | Xyl-Glc | COOH | OH | CH_3_ | CH_3_ | H | β-CH_3_ | β-CH_3_ | α-H | CH_3_ |
|  | Ilexsaponin B_3_( Hidaka *et al*., 1987) | Xyl –Glc | COOGlc | OH | CH_3_ | CH_3_ | H | β-CH_3_ | β-CH_3_ | α-H | CH_3_ |
|  | Ilexsaponin B_4_(Feng *et al*., 2008b) | Ara-Glc-Xyl | COOH | OH | CH_3_ | CH_3_ | H | β-CH_3_ | α-CH_3_ | β-H | CH_3_ |
|  | Ilexpublesnin K(Zhou *et al*., 2013) | Ara-Glc-Xyl | COOGlc | OH | CH_3_ | CH_3_ | H | β-CH_3_ | β-CH_3_ | α-H | CH_3_ |
| type | Name | R_1_ | R_2_ | R_3_ | R_4_ | R_5_ | R_6_ | R_7_ | R_8_ | R_9_ | R_10_ |
| B | Ilexpublesnin J(Zhou *et al*., 2013) | Glc-Glc-Ara | COOGlc | OH | CH_3_ | CH_3_ | H | β-CH_3_ | β-CH_3_ | α-H | CH_3_ |
|  | Ilexpublesnin O(Zhou *et al*., 2014) | Glc-Glc-Xyl | COOH | OH | CH_3_ | CH_3_ | H | β-CH_3_ | β-CH_3_ | α-H | CH_3_ |
|  | Ilexpublesnin N(Zhou *et al*., 2014) | Rha-Glc-Xyl | COOGlc | H | CH_3_ | CH_3_ | H | β-CH_3_ | α-CH_3_ | β-OH | CH_3_ |
|  | Ilexoside O(Han *et al*., 1987) | Rha-Glc-Xyl | COOGlc | OH | CH_3_ | CH_3_ | H | β-CH_3_ | β-CH_3_ | α-H | CH_3_ |
|  | Ilexpublesnin E(Zhou *et al*., 2013) | Rha-Glc-Xyl | COOGlc | OH | CH_3_ | CH_3_ | H | β-CH_3_ | α-CH_3_ | β-H | CH_3_ |
|  | Ilexoside P(Zhou *et al*., 2013c) | Rha-Glc-Xyl | COOGlc | OH | CHO | CH_3_ | H | β-CH_3_ | α-CH_3_ | β-H | CH_3_ |
|  | Ilexsaponin B_2_( Hidaka *et al*., 1987) | Xyl-Glc-Rha | COOH | OH | CH_3_ | CH_3_ | H | β-CH_3_ | β-CH_3_ | α-H | CH_3_ |
| D | Heterobetulinic acid 3-O-β-glucopyranosyl（1→2）-β-D-xylopyranoside(Wu *et al*., 2012) | Glc-Xyl | COOH |  |  |  |  |  |  |  |  |
| E | Taraxerol (Feng, 2012) | H |  |  |  |  |  |  |  |  |  |
| F | Ilexsaponin C(Lin *et al*., 2011) | Glc-Xyl | β-CH_3_ | α-CH_3_ |  |  |  |  |  |  |  |
| G | Ilexsaponin F(Li *et al*., 2012) | Ara-Glc | COOGlc | β-CH_3_ | β-CH_2_OH | COOH |  |  |  |  |  |
| H | Ilexodic acid(Zhang *et al*., 1983) | H | COOH | β-CH_3_ | β-CH_3_ | α-H | CH_3_ | CH_3_ | CH_3_ | H |  |
|  | Ilexsaponin B(Jiang *et al*., 1991) | Xyl | COOGlc | β-CH_3_ | β-CH_3_ | α-H | CH_3_ | CH_3_ | CH_3_ | H |  |
|  | Ilexsaponin D(Li *et al*., 2012) | Ara-Glc | COOGlc | β-CH_3_ | α-CH_3_ | β-H | CH_3_ | CH_3_ | CH_3_ | H |  |
|  | Ilexsaponin E(Li *et al*., 2012) | Ara-Glc | COOH | β-CH_3_ | α-CH_3_ | β-H | CH_3_ | CH_3_ | CH_3_ | H |  |
|  | Ilexsaponin G(Li *et al*., 2014) | Ara-Glc | COOGlc | β-CH_3_ | α-CH_3_ | β-H | CH_3_ | COOH | CH_3_ | H |  |
|  | Ilexsaponin H(Li *et al*., 2014) | Ara-Glc | COOH | β-CH_3_ | α-CH_3_ | β-H | CH_3_ | COOH | CH_3_ | H |  |
|  | Ilexpublesnin F(Zhou *et al*., 2013) | Glc-Xyl | COOH | β-CH_3_ | β-CH_3_ | α-H | CH_3_ | CH_3_ | CH_3_ | H |  |
|  | Ilexolide H(Han *et al*., 1987) | Glc-Xyl | COOGlc | β-CH_3_ | β-CH_3_ | α-H | CH_3_ | CH_3_ | CH_3_ | H |  |
|  | Pubescenoside C(Wang *et al*., 2008) | Glc-Xyl | COOGlc | γ-CH_3_ | β-CH_3_ | α-H | CH_3_ | CH_3_ | CH_3_ | H |  |
|  | Ilexpublesnin T(Wu *et al*., 2015) | Rha-Glc-Xyl | COOH | γ-CH_3_ | β-CH_3_ | α-H | CH_3_ | CH_3_ | CH_3_ | H |  |
|  | Pubescenoside D(Wang *et al*., 2008) | Rha-Glc-Xyl | COOGlc | γ-CH_3_ | β-CH_3_ | α-H | CH_3_ | CH_3_ | CH_3_ | H |  |

* The triterpenoid skeleton configurations are corresponded to Figure S8. Glc = D-glucopyranose, xyl = D-xylopyranose, rha = L-rhamnopyranose, ara = L-arabinose, GlcA = glucuronic acid and γ-R (solid), β-R (wedge solid) and α-R (wedge dotted) means the bond is in, upwardly extending and downwardly projecting the paper, and wavy line represents a bond can be in one of the three positions. The gray portion indicates that the compound belongs to β-amyrin type.

**References:**

Arthur, H. R., Lee, C. M., and Ma, C. N. (1956). 295. The occurrence of triterpenes in the aquifoliaceae and ericaceae of Hong Kong. *Journal of the Chemical Society* 9,1461-1463.

Cai, X., Liu, Z. Q., Zhu, C. C., Wang, P. X. and Liu, L. (2001). Advances in chemical composition, pharmacologic action and clinical application of *Ilex pubescens*. *Guangdong Pharmaceutial Journal*(in Chinese) 11, 4-6.

Feng, F., Zhu, M. X., and Xie, N. (2008). Studies on the Chemical Constituents of the Roots of *Ilex pubescens*. *Chinese Pharmaceutical Journal* (in Chinese) 43, 732-736. doi: 10.3321/j.issn:1001-2494.2008.10.004.

Feng, F., Zhu, M. X., Xie, N., Liu, W. Y., Chen, D. J., and You, Q. D. (2008b). Two new triterpenoid saponins from the root of *Ilex pubescens*. *J Asian Nat Prod Res* 10, 71-75. doi: 10.1080/10286020701273874.

Feng, H. F. (2012). Studies on chemical constituents of *Ilex pubescens* leaves: *Guangxi Normal University* (in Chinese).

Han, Y. N., Baik, S. K., Kim, T. K., and Han, B. H. (1987). Antithrombotic activities of saponins from *llex pubescens*. *Arch Pharm* 10, 115-120.

Hidaka, K., Ito, M., Matsuda, Y., Kohda, H., Yamasaki, K., and Yamahara, J. (1986). A triterpene and saponin from roots of *Ilex pubescens*. *Phytochemistry* 26, 2023-2027.

Hidaka, K., Ito, M., Matsuda, Y., Kohda, H., Yamasaki, K., Yamahara, J., *et al.* (1987). New triterpene saponins from *Ilex pubescens*. *Chemical and Pharmaceutical Bulletin* 35, 524-529.

Jiang, Y. P., Feng, F., Xie. N., Cheng. L., and Zhu, M. X. (2008). Chemical constituents from the root of *Ilex pubescens* Hook. *Pharmaceutical and Clinical Research*(in Chinese) 16, 163-165.

Jiang, Z. F. and Huang, R. X. (1991). Studies on Chemical Constituents of *Ilex pubescen*s: Ⅲ. Separation and Identification of Four Triterpenoid Saponins. Chinese herbal medicine 22, 291-294.

Li, L., He, Y. X., Gou, M. L. and Dai, C. (2012). Three new triterpenoid saponins from *Ilex pubescens*. *J Asian Nat Prod Res* 14, 1169-1174. doi: 10.1080/10286020.2012.738674.

Li, L., Feng, L. S. and He, Y. X. (2014). Cytotoxic triterpenesaponins from *Ilex pubescens*. *J Asian Nat Prod Res* 16, 830-5. doi: 10.1080/10286020.2014.920012.

Lin, L., P., Qu, W., and Liang, J. Y. (2011). Triterpene saponins with XOD inhibitory activity from the roots of *Ilex pubescens*. *Chinese Chemical Letters*, 22, 697-700.

Lin, L. P., Li, Y., Zhang, J. C., and Lu, Y. (2015). Biomarkers for *Ilex pubescens* under anti-platelet activity-oriented isolation. *Biochem Syst Ecol* 61, 8-11. doi: 10.1016/j.bse.2015.05.006.

Wang, J. R., Zhou, H., Jiang, Z. H. and Liu, L. (2008). Two new triterpene saponins from the anti-inflammatory saponin fraction of *Ilex pubescens* root. *Chem Biodivers* 5, 1369-76. doi: 10.1002/cbdv.200890125.

Wu, P., Gao, H., Li, Z.H. and Liu, Z.Q. (2015). Two new triterpene saponins from the roots of *Ilex pubescens*. *Phytochem Lett* 12, 17-21. doi: 10.1016/j.phytol.2015.02.015.

Wu, T., Zhang, Q. W., Zhang, X. Q., Liu, G., Wang, L., Jiang, M. M., *et al.* (2012). Two new compounds from the roots of *Ilex pubescens*. *Nat Prod Res* 26, 1408-12. doi: 10.1080/14786419.2011.598155.

Zhang, C. X., Lin, C. Z., Xiong, T. Q., Zhu, C. C., Yang, J. Y., and Zhao, Z. X. (2010). New triterpene saponins from the root of *Ilex pubescens*. *Fitoterapia* 81, 788-792. doi: 10.1016/j.fitote.2010.04.008.

Zhang, C. X., Lin, C. Z., Yang, J. Y., Xiong, T. Q., Deng, J. W., Wei, Y. L., *et al.* (2011). Triterpene saponins isolated from *Ilex pubescens*(III). *ACTA Scientiarum Naturalium Universitatis Sunyatseni*(in Chinese) 50, 70-74.

Zhang, S. D., Zeng, L. M., and Su, J. Y. (1983). Study on the structure of Ilexodic acid. *Chemistry Bulletin*(in Chinese) 15-16.

Zhao, Z. X., Jin, J., Lin, C. Z., Xiong, T. Q., Luo, H. F., Qin, C. Y., *et al.* (2011). Study on triterpenoid glycosides from roots of *Ilex pubescens*. *China Pharmacist* (in Chinese) 14, 599-601.

Zhao, Z. X., Zeng, R. X., Jin, J., Lin, C. Z., Xiong, T. Q., Cai, J. Y., *et al.* (2012). A new triterpenoid glycoside from roots of *Ilex pubescens*. *Chinese Traditional and Herbal Drugs*(in Chinese) 43, 1267-1269.

Zhou, Y., Zhou, S. X., Jiang, Y., Sun, J., and Tu, P. F. (2012). Chemical constituents in leaves of *Ilex pubescens*. *Chinese Traditional and Herbal Drug* (in Chinese) 43, 1479-1483.

Zhou, Y., Chai, X. Y., Zeng, K. W., Zhang, J. Y., Li, N., Jiang, Y., *et al.* (2013). Ilexpublesnins C-M, eleven new triterpene saponins from the roots of *Ilex pubescens*. *Planta Med* 79, 70-7. doi: 10.1055/s-0032-1327927.

Zhou, Y., Zeng, K., Zhang, J., Li, N., Chai, X., Jiang, Y., *et al.* (2014). Triterpene saponins from the roots of *Ilex pubescens*. *Fitoterapia* 97, 98-104. doi: 10.1016/j.fitote.2014.05.020.

Zhou, Z. L., Feng, Z. C., Yin, W. Q., and Zhang, H. L. (2013b). A new triterpene saponin from the leaves of *Ilex pubescens*, and its XOD inhibitory activity. *Chemistry of Natural Compounds* 49, 682-684.

Zhou, Z. L., Feng, Z. C., Fu, C. Y. and Zeng, L. (2013c). A new triterpene saponin from the roots of *Ilex pubescens*. *Nat Prod Res* 27, 1343-7. doi: 10.1080/14786419.2012.738208.
