# Supplementary material for: Transcriptomic Comparison Reveals Candidate Genes for Triterpenoid Biosynthesis in Two Closely Related Ilex Species
Source: Front Plant Sci. 2017 Apr 28;8:634. doi: 10.3389/fpls.2017.00634 (PMC5408325; doi:10.3389/fpls.2017.00634)
Supplement: Table S2 — Identified pentacyclic triterpenoids in I. asprella. [file Table2.DOC]

**Table S2. Identified pentacyclic triterpenoids** **in** ***I. asprella***

| type | Name | R_1_ | R_2_ | R_3_ | R_4_ | R_5_ | R_6_ | R_7_ | R_8_ | R_9_ | R_10_ |
| --- | --- | --- | --- | --- | --- | --- | --- | --- | --- | --- | --- |
| A | 3β-O- acetyluvaol(He *et al*., 2012) | COCH_3_ | COOH | H | H |  |  |  |  |  |  |
|  | 3β-O- acetylursolic acid(He *et al*., 2012) | COCH_3_ | COOH | H | OH |  |  |  |  |  |  |
| B | Ursolic acid(Huang, 2011) | H | COOH | H | CH_3_ | CH_3_ | H | β-CH_3_ | α-CH_3_ | β-H | CH_3_ |
|  | Uvaol acetate(Huang, 2011) | COCH_3_ | CH_2_OH | H | CH_3_ | CH_3_ | H | β-CH_3_ | α-CH_3_ | β-H | CH_3_ |
|  | Ilexoside XXIX(Cai *et al*., 2010) | SO_3_Na | COOGlc | OH | CH_3_ | CH_3_ | H | β-CH_3_ | β-CH_3_ | α-H | CH_3_ |
|  | Monepaloside F(Cai *et al*., 2010) | Xyl | COOGlc | OH | CH_3_ | CH_3_ | H | β-CH_3_ | α-CH_3_ | β-H | CH_3_ |
|  | Ilexasprellanoside D(Yu *et al*., 2014) | H | COOGlc | OH | CH_3_ | COOH | H | β-H | β-CH_3_ | α-CH_3_ | CH_3_ |
|  | Ilexasprellanoside E(Yu *et al*., 2014) | 2’-*O*-Ac-Xyl | COOH | OH | CH_3_ | CH_3_ | H | β-H | β-CH_3_ | α-CH_3_ | CH_3_ |
|  | Ilexasprellanoside F(Yu *et al*., 2014) | GlcA | COOH | OH | CH_3_ | CH_3_ | H | β-H | β-CH_3_ | α-CH_3_ | CH_3_ |
|  | 2α,3β,19α-trihydroxy-urs-12-ene-23,28-dioic acid(Wang, 2008) | H | COOH | OH | CH_3_ | COOH | OH | β-CH_3_ | α-CH_3_ | β-H | CH_3_ |
|  | Rotundioic acid(Wang, 2008) | H | COOH | OH | CH_3_ | COOH | H | β-CH_3_ | α-CH_3_ | β-H | CH_3_ |
|  | Ilexgenin A(Zhou *et al*., 2012) | H | COOH | OH | COOH | CH_3_ | H | β-CH_3_ | α-CH_3_ | β-H | CH_3_ |
|  | Pomolic acid(Huang, 2011) | H | COOH | OH | CH_3_ | CH_3_ | H | β-CH_3_ | α-CH_3_ | β-H | CH_3_ |
|  | 28-O-β-D-glucopyranosyl pomolic acid(Cai *et al*., 2010) | H | COOGlc | OH | CH_3_ | CH_3_ | H | β-CH_3_ | α-CH_3_ | β-H | CH_3_ |
|  | Ilexsaponin A_1_(Cai *et al*., 2010) | H | COOGlc | OH | COOH | CH_3_ | H | β-CH_3_ | α-CH_3_ | β-H | CH_3_ |
|  | 2α,3β,19α-trihydroxy-urs-12-ene-24,28-dioic-28-O-β-D-glucopyranoside(Wang, 2008) | H | COOGlc | OH | COOH | CH_3_ | OH | β-CH_3_ | α-CH_3_ | β-H | CH_3_ |
|  | 3β,19α-dihydroxyolean-12-ene-24,28-dioicacid-28-O-β-D-glucopyranoside(Wang *et al*.,2014) | H | COOGlc | OH | COOH | CH_3_ | H | α-H | β-CH_3_ | α-CH_3_ | CH_3_ |
|  | 3-O-β-sulfooxy-19-hydroxyurs-12-ene-28-oicacid(Wang *et al*., 2014) | SOH_3_ | COOH | OH | CH_3_ | CH_3_ | H | β-CH_3_ | α-CH_3_ | β-H | CH_3_ |
|  | Ilexoside B(Cai *et al*., 2010) | Xyl | COOH | OH | CH_3_ | CH_3_ | H | β-CH_3_ | α-CH_3_ | β-H | CH_3_ |
|  | Oblonganoside I(Zhou *et al*., 2012) | Xyl | COOGlc | OH | CH_3_ | CH_2_OH | H | β-CH_3_ | α-CH_3_ | β-H | CH_3_ |
|  | Oblonganoside H(Wang *et al*., 2009) | Xyl | COOGlc | OH | CH_3_ | CH_3_ | H | β-CH_3_ | α-CH_3_ | β-H | CH_3_ |
|  | Asprellanoside A(Zhou *et al*., 2012) | Sulphonyl-Xyl | COOGlc | OH | CH_3_ | CH_3_ | H | β-CH_3_ | α-CH_3_ | β-H | CH_3_ |
|  | tormentoside(Wang *et al*., 2014) | H | COOGlc | OH | CH_3_ | CH_3_ | OH | β-CH_3_ | α-CH_3_ | β-H | CH_3_ |
| type | Name | R_1_ | R_2_ | R_3_ | R_4_ | R_5_ | R_6_ | R_7_ | R_8_ | R_9_ | R_10_ |
| B | Ilexsaponin B_2_(Kashiwada *et al*., 1993) | Xyl-Glc-Rha | COOH | OH | CH_3_ | CH_3_ | H | β-CH_3_ | α-CH_3_ | β-H | CH_3_ |
|  | Ilexasprellanoside C(Yu *et al*., 2014) | GlcA | COOH | OH | CH_3_ | CH_3_ | H | β-CH_3_ | α-CH_3_ | β-H | CH_3_ |
|  | Ilexasoside A(Wang *et al*., 2012) | GlcA-5’-COOCH_3_ | COOH | OH | CH_3_ | CH_3_ | H | β-CH_3_ | α-CH_3_ | β-H | CH_3_ |
|  | Ilexasoside D(Wang *et al*., 2012) | GlcA-3’-OSO_3_Na | COOGlc | OH | CH_3_ | CH_3_ | H | β-CH_3_ | α-CH_3_ | β-H | CH_3_ |
|  | Ilexasoside C(Wang *et al*., 2012) | GlcA-5’-COOCH_3_ | COOGlc | OH | CH_3_ | CH_3_ | H | β-CH_3_ | α-CH_3_ | β-H | CH_3_ |
|  | Ilexasoside B(Wang *et al*., 2012) | GlcA-3’-OSO_3_Na | COOH | OH | CH_3_ | CH_3_ | H | β-CH_3_ | α-CH_3_ | β-H | CH_3_ |
|  | Ziyu-glycosides Ⅰ(Li *et al*., 1997a) | Ara | COOGlc | OH | CH_3_ | CH_3_ | H | β-CH_3_ | α-CH_3_ | β-H | CH_3_ |
|  | 3β-[(α-L-arabinopyranosyl)oxy]-19α-hydroxy-urs-12-en-28-oic acid 28-β-D-glucopyranosyl ester(Wang, 2008) | Ara | COO-6-O-Galloyl-Glc | OH | CH_3_ | CH_3_ | H | β-CH_3_ | α-CH_3_ | β-H | CH_3_ |
|  | Ilexside I(Zhou *et al*., 2012) | Ara-Glc | COOH | OH | CH_3_ | CH_3_ | H | β-CH_3_ | α-CH_3_ | β-H | CH_3_ |
|  | Ilexasprellanoside B(Yu *et al*., 2014) | 2’-O-Ac-xyl | COOH | OH | CH_3_ | CH_3_ | H | β-CH_3_ | α-CH_3_ | β-H | CH_3_ |
|  | Asprellanosides B(Zhou *et al*., 2012) | Sulfur-Ara | COOH | OH | CH_3_ | CH_3_ | H | β-CH_3_ | β-CH_3_ | α-H | CH_3_ |
|  | 18-dehydrouraolic acid(Huang, 2011) | H | COOH | H | CH_3_ | CH_3_ | H | β-CH_3_ | α-CH_3_ | β-H | CH_3_ |
|  | Suavissimoside R_1_(Wang *et al*.,2014) | H | COOGlc | OH | CH_3_ | COOH | OH | β-H | α-CH_3_ | β-H | CH_3_ |
|  | 2α,3β,19α-trihydroxy-olean-12-ene-23,28,dioic acid(Wang, 2008) | H | COOH | OH | CH_3_ | COOH | OH | β-H | α-CH_3_ | β-CH_3_ | CH_3_ |
|  | 3*β*-[(*α*-L-arabinopyranosyl)oxy]-19α-hydro-xyolean-12-en-28-oic acid-28-β-D-glucopyranosyl ester(Wang, 2008) | Ara | COOGlc | OH | CH_3_ | CH_3_ | H | β-H | *α*-CH_3_ | β-CH_3_ | CH_3_ |
|  | Ilexoside E(Wang, 2008) | H | COOGlc | OH | COOH | CH_3_ | H | β-H | *α*-CH_3_ | β-CH_3_ | CH_3_ |
|  | Oleanolic acid(He *et al*., 2012) | H | COOH | H | CH_3_ | CH_3_ | H | β-H | *α*-CH_3_ | β-CH_3_ | CH_3_ |
|  | Oleanolic acid-3-*O-β*-glucuronopyranoside(Lei, 2008) | Glc | COOH | H | CH_3_ | CH_3_ | H | β-H | *α*-CH_3_ | β-CH_3_ | CH_3_ |
|  | Asprellic acid A(Kashiwada *et al*., 1993) | *Trans*-Coum | COOH | H | CH_3_ | CH_3_ | H | β-H | *α*-CH_3_ | β-CH_3_ | CH_2_O-  *trans*-Coum |
|  | Asprellic acid B(Kashiwada *et al*., 1993) | *Trans*-Coum | COOH | H | CH_3_ | CH_3_ | H | β-H | *α*-CH_3_ | β-CH_3_ | CH_2_O-*cis*  -Coum |
| type | Name | R_1_ | R_2_ | R_3_ | R_4_ | R_5_ | R_6_ | R_7_ | R_8_ | R_9_ | R_10_ |
| B | Asprellic acid C(Kashiwada *et al*., 1993) | *Cis-*-Coum | COOH | H | CH_3_ | CH_3_ | H | β-H | *α*-CH_3_ | β-CH_3_ | CH_2_O- *trans*-Coum |
| C | Ilexasoside H(Wang *et al*., 2012) | GlcA -5’-COOCH_3_ | COOGlc |  |  |  |  |  |  |  |  |
|  | Asprellanosides C(Zhang *et al*., 2013) | Sulfur-Xyl | COOGlc |  |  |  |  |  |  |  |  |
| F | Ilexoside H(Wang *et al*., 2012) | Xyl | β-CH_3_ | α-CH_3_ |  |  |  |  |  |  |  |
|  | 28-Nor-19βH,20αH-Ursa-12,17-dien-3-ol(Huang, 2011) | H | α-CH_3_ | β-CH_3_ |  |  |  |  |  |  |  |
| G | 19-dehydrouraolic acid(Li *et al*., 1997) | H | COOH | γ-CH_3_ | γ-CH_3_ | CH_3_ |  |  |  |  |  |
|  | Oblonganoside B(Cai *et al*., 2010b) | Xyl | COOGlc | γ-CH_3_ | γ-CH_3_ | CH_3_ |  |  |  |  |  |
|  | Ilexasoside G(Wang *et al*., 2012) | GlcA-5’-COOCH_3_ | COOGlc | γ-CH_3_ | γ-CH_3_ | CH_3_ |  |  |  |  |  |
|  | Asprellanosides D(Zhang *et al*., 2013) | Sulfur-Xyl | COOGlc | γ-CH_3_ | γ-CH_3_ | CH_3_ |  |  |  |  |  |
|  | Asprellanosides E(Zhang *et al*., 2013) | Sulfur-Xyl | COOH | γ-CH_3_ | γ-CH_3_ | CH_3_ |  |  |  |  |  |
| H | Ilexsaponin B(Cai *et al*., 2010) | Xyl | COOGlc | β-CH_3_ | β-CH_3_ | *α*-H | CH_3_ | CH_3_ | CH_3_ | H |  |
|  | Ilexasoside E(Wang *et al*., 2012) | GlcA-5’-COOCH_3_ | COOGlc | γ-CH_3_ | *α*-CH_3_ | β-H | CH_3_ | CH_3_ | CH_3_ | H |  |
|  | Ilexasprellanoside A(Yu *et al*., 2014) | Xyl | COOH | γ-CH_3_ | *α*-CH_3_ | β-H | CH_3_ | CH_3_ | CH_3_ | H |  |
|  | Ilexolic acid(Cai *et al*., 2010) | H | COOH | β-CH_3_ | β-CH_3_ | *α*-H | CH_3_ | CH_3_ | CH_3_ | H |  |
|  | Ilexasoside F(Wang *et al*., 2012) | GlcA-5’-COOCH_3_ | COOGlc | γ-CH_3_ | β-CH_3_ | *α*-H | CH_3_ | CH_3_ | CH_3_ | H |  |
|  | 3-O-β-D-xylopyranosyl-3β-hydroxyurs-12,18(19)-dien-28-oic acid 28-β-D-glucopyranosyl ester(Cai *et al*., 2010b) | Xyl | COOGlc | γ-CH_3_ | *α*-CH_3_ | β-H | CH_3_ | CH_3_ | CH_3_ | H |  |
|  | Randialic acid B(Cai *et al*., 2010) | H | COOH | γ-CH_3_ | *α*-CH_3_ | β-H | CH_3_ | CH_3_ | CH_3_ | H |  |
| I | Asprellols A(Jiang *et al*., 2014) | CH_3_ | CH_3_ | OH |  |  |  |  |  |  |  |
|  | Asprellols B(Jiang *et al*., 2014) | CH_3_ | CH_2_ | CH_2_ |  |  |  |  |  |  |  |
|  | Asprellols C(Jiang *et al*., 2014) | H | CH_3_ | CH_3_ |  |  |  |  |  |  |  |
|  | 2,6β-dihydroxy-3-oxo-11α,12α-epoxy-24-norursa-1,4-dien-28,13β-olide(Jiang *et al*., 2014) | CH_3_ | CH_3_ | H |  |  |  |  |  |  |  |

* The triterpenoid skeleton configurations are corresponded to Figure S8. Glc = D-glucopyranose, xyl = D-xylopyranose, rha = L-rhamnopyranose, ara = L-arabinose, GlcA = glucuronic acid, Coum = coumarate and γ-R (solid), β-R (wedge solid) and α-R (wedge dotted) means the bond is in, upwardly extending and downwardly projecting the paper, and wavy line represents a bond can be in one of the three positions. The gray portion indicates that the compound belongs to β-amyrin type.

**References:**

Cai, Y., Zhang, Q. W., Li, Z. J., Fang, C. L., Wang, L., Zhang, X. Q., *et al.* (2010)., Chemical constituents from roots of *Ilex asprella*(in Chinese). 41, 1426-1429.

Feng, F., Zhu, M. X., and Xie, N. (2008). Studies on the Chemical Constituents of the Roots of *Ilex pubescens*. *Chinese Pharmaceutical Journal* (in Chinese) 43, 732-736. doi: 10.3321/j.issn:1001-2494.2008.10.004.

He, W. J., Zhao, Z. X., Lin, C. Z., and Zhu. C. C. (2012). Study on triterpenoids from the roots of *Ilex asprella*. *West China Journal of Pharmaceutcal Sciences*(in Chinese) 27, 51-53.

Huang, J. C. (2011). Studies on chemical composition and the quality of Gangmei: *Guangzhou university of Chinese Medcine* (in Chinese).

Jiang, K., Bai, J. Q., Chang, J., and Tan, J. J. (2014). Three New 24-Nortriterpenoids from the Roots of *Ilex asprella*. *Helv Chim Acta* 97, 64-69.

Kashiwada, Y., Zhang, D. C., Chen, Y. P., Cheng, C. M., Chen, H. T. Chang, H. C., *et al.* (1993). Antitumor agents, 145. Cytotoxic asprellic acids A and C and asprellic acid B. new p-coumaroyl triterpenes, from *Ilex asprella*. *J Nat Prod* 56, 2077-82.

Lei, Y. (2008). Studies on the Chemical Constituents and Bioactivities of *Ilex asprella* and *Miliusa balaeca*, in: Shenyang Pharmaceutical University(in Chinese).

Li, M. H., Yu, S. J., and Du, S. J. (1997). Studies on the chemical constituents of the root of roughhaired holly (*Ilex asprella*). *Chin Tradit Herb Drugs*(in Chinese) 28, 454 -456.

Wang, C. and Tu, P. (2014). Chemical constituents from the leaves of *Ilex asprella* (Hook. et Arn.) Champ. ex Benth. *Journal of Chinese Pharmaceutical Sciences* 23. doi: 10.5246/jcps.2014.11.099.

Wang, H. L. (2008). Study on Chemical constituents of the leaves of *Ilex asprella*: *Shenyang Pharmaceutical University* (in Chinese).

Wang, H. L., Wu. L. J., Lei, Y., Zhou, S. X., and Tu, P. F. (2009). Chemical constituents of the leaves of *Ilex asprella*. *Journal of Shenyang Pharm aceutical University*(in Chinese) 26, 279-281+298.

Wang, L., Cai, Y., Zhang, X. Q., Fan, C. L., Zhang, Q. W., Lai, X. P., *et al.* (2012). New triterpenoid glycosides from the roots of *Ilex asprella*. *Carbohyd Res* 349, 39-43. doi: 10.1016/j.carres.2011.12.003.

Yu, L., Shi, S.P., Song, Y.L., Bi, D. and Tu, P.F. (2014). Triterpene saponins from the roots of *Ilex asprella*. *Chem Biodivers* 11, 767-75. doi: 10.1002/cbdv.201300155.

Zhang, Z. X., Fu, Q., and Zheng, K. Y. (2013). Three new triterpene glycosides from *Ilex asprella*. *J Asian Nat Prod Res* 15, 453-8. doi: 10.1080/10286020.2013.783023.

Zhou, M., Xu, M., Ma, X. X., Zheng, K., Yang, K., Yang, C. R., *et al.* (2012). Antiviral triterpenoid saponins from the roots of *Ilex asprella*. *Planta Med* 78, 1702-1705. doi: 10.1055/s-0032-13152.
