# Supplementary material for: Transcriptomic Comparison Reveals Candidate Genes for Triterpenoid Biosynthesis in Two Closely Related Ilex Species
Source: Front Plant Sci. 2017 Apr 28;8:634. doi: 10.3389/fpls.2017.00634 (PMC5408325; doi:10.3389/fpls.2017.00634)
Supplement: Table S3 — Information of Characterized CYPs and UGTs for Blastp analysis. [file Table3.DOC]

| Key enzymes | Name | Origin | GenBank accession number |
| --- | --- | --- | --- |
| CYP | CYP51H10 | *Avena strigosa* | ABG88965.1 |
|  | CYP716A1 | *Arabidopsis lyrata* | EFH44718.1 |
|  | CYP716A2 | *Arabidopsis thaliana* | NP_198463.1 |
|  | CYP716A12 | *Medicago truncatula* | ABC59076.1 |
|  | CYP716A14v2 | *Artemisia annua* | AHF22083.1 |
|  | CYP716A15 | *Vitis vinifera* | NP_001268115.1 |
|  | CYP716A17 | *V. vinifera* | NP_001268076.1 |
|  | CYP716A52v2 | *Panax ginseng* | I7C6E8.1 |
|  | CYP716A75 | *Maesa lanceolata* | AHF22088.1 |
|  | CYP716AL1 | *Catharanthus roseus* | AEX07773.1 |
|  | CYP716Y1 | *Bupleurum falcatum* | AHF45909.1 |
|  | CYP72A61V2 | *M. truncatula* | BAL45199.1 |
|  | CYP72A68V2 | *M. truncatula* | BAL45204.1 |
|  | CYP72A63 | *M. truncatula* | BAL45200.1 |
|  | CYP72A154 | *Glycyrrhiza uralensis* | BAL45207.1 |
|  | CYP87D16 | *Maesa lanceolata* | AHF22090.1 |
|  | CYP88D6 | *Glycyrrhiza uralensis* | B5BSX1.1 |
|  | CYP93E1 | *Glycine max* | NP_001236154.1 |
|  | CYP93E2 | *M. truncatula* | ABC59085.1 |
|  | CYP93E3 | *Glycyrrhiza uralensis* | BAG68930.1 |
|  | CYP93E4 | *Arachis hypogaea* | AIN25416.1 |
|  | CYP93E5 | *Cicer arietinum* | AIN25417.1 |
|  | CYP93E6 | *Glycyrrhiza glabra* | AIN25418.1 |
|  | CYP93E7 | *Lens culinaris* | AIN25419.1 |
|  | CYP93E8 | *Pisum sativum* | AIN25420.1 |
|  | CYP93E9 | *Phaseolus vulgaris* | AIN25421.1 |
| UGT | UGT73C10 | *Barbarea vulgaris* | AFN26666.1 |
|  | UGT73C11 | *B. vulgaris* | AFN26667.1 |
|  | UGT73C12 | *B. vulgaris* | AFN26668.1 |
|  | UGT73C13 | *B. vulgaris* | AFN26669.1 |
|  | UGT74M1 | *G. max* | AB473731.1 |
|  | UGT73F2 | *G. max* | BAM29362.1 |
|  | UGT73F3 | *M. truncatula* | ACT34898.1 |
|  | UGT73F4 | *G. max* | BAM29363.1 |
|  | UGT73P2 | *G. max* | AB473730.1 |

**Table S3**. Information of Characterized CYPs and UGTs for Blastp analysis
