# Supplementary material for: Transcriptomic Comparison Reveals Candidate Genes for Triterpenoid Biosynthesis in Two Closely Related Ilex Species
Source: Front Plant Sci. 2017 Apr 28;8:634. doi: 10.3389/fpls.2017.00634 (PMC5408325; doi:10.3389/fpls.2017.00634)
Supplement: Table S4 — Designed primers for IpAS1 cloning. [file Table4.DOC]

**Table S4. Designed primer for IpAS1 clone**

| Primer ID | Primer sequence | Length/bp |
| --- | --- | --- |
| IpAS1-F | TGAAAATTCGAATTCATGTGGAAGCTTAAGATTGCTG | 37 |
| IpAS1-R1 | TTAATGATGATGATGATGATGGACATTCTGGGAAGGTGACC | 41 |
| IpAS1-R2 | GAATTGTTAATTAAGAGCTCTTAATGATGATGATGATGATGGACATTCTGGGAAGGT | 57 |
