# Supplementary material for: Transcriptomic Comparison Reveals Candidate Genes for Triterpenoid Biosynthesis in Two Closely Related Ilex Species
Source: Front Plant Sci. 2017 Apr 28;8:634. doi: 10.3389/fpls.2017.00634 (PMC5408325; doi:10.3389/fpls.2017.00634)
Supplement: Table S5 — The CYPs with length >1,000 bp and classified as CYP72 clan and CYP85 clan in I. pubescens. [file Table5.DOC]

**Table S5 The CYPs which length > 1000 bp and classified as CYP72 clan and CYP85 clan in *I. pubescens***

| clan | name | Length/bp | RPKM | clan | name | Length/bp | RPKM |
| --- | --- | --- | --- | --- | --- | --- | --- |
| CYP72 | Unigene0032611 | 1745 | 27.45 | CYP85 | Unigene0038563 | 1185 | 3.55 |
|  | Unigene0024642 | 2316 | 107.59 |  | Unigene0036174 | 1712 | 76.76 |
|  | Unigene0043584 | 1791 | 17.99 |  | Unigene0036170 | 1727 | 72.57 |
|  | Unigene0027359 | 1723 | 9.45 |  | Unigene0036172 | 2116 | 98.78 |
| CYP85 | Unigene0024294 | 1726 | 14.67 |  | Unigene0048478 | 1833 | 75.33 |
|  | Unigene0016993 | 1621 | 3.25 |  | Unigene0036171 | 1878 | 25.47 |
|  | Unigene0031098 | 1032 | 25.28 |  | Unigene0026902 | 1085 | 1.70 |
