# Supplementary material for: Transcriptomic Comparison Reveals Candidate Genes for Triterpenoid Biosynthesis in Two Closely Related Ilex Species
Source: Front Plant Sci. 2017 Apr 28;8:634. doi: 10.3389/fpls.2017.00634 (PMC5408325; doi:10.3389/fpls.2017.00634)
Supplement: Table S6 — Putative Ilex-genus-specific CYPs. [file Table6.DOC]

**Table S6.** Putative Ilex-genus-specific *CYP*s

| OrthMCL | *I. pubescens* | | | *I. asprella* | | | | |
| --- | --- | --- | --- | --- | --- | --- | --- | --- |
|  | Name | Length | RPKM | Name | | Length | | RPKM |
| 100 | Unigene0048478 | 1833 | 75.33 | CL1693.Contig1 | | 1983 | | 127.81 |
| 101 | Unigene0051102 | 305 | 0.58 | CL2312.Contig2 | | 321 | | 2.55 |
| 102 | Unigene0049493 | 1912 | 224.15 | CL232.Contig1 | | 2171 | | 140.30 |
| 103 | Unigene0043247 | 1313 | 8.57 | CL271.Contig1 | | 1278 | | 88.41 |
| 104 | Unigene0052249 | 1221 | 2.13 | CL2897.Contig2 | | 1749 | | 11.55 |
| 105 | Unigene0045337 | 1567 | 19.15 | CL6196.Contig4 | | 1760 | | 45.48 |
| 106 | Unigene0003417 | 1792 | 2.42 | Unigene989 | | 2311 | | 23.33 |
| 115 | Unigene0013355 | 465 | 1.38 | Unigene24054 | | 1006 | | 9.32 |
| 117 | Unigene0018567 | 1248 | 4.38 | Unigene20974 | | 1999 | | 87.25 |
| 119 | Unigene0024880 | 1792 | 6.61 | Unigene10536 | | 1784 | | 14.53 |
| 120 | Unigene0026130 | 1937 | 154.85 | Unigene3887 | | 1614 | | 21.16 |
| 121 | Unigene0027209 | 632 | 16.89 | Unigene24861 | | 479 | | 13.56 |
| 122 | Unigene0028539 | 597 | 144.35 | Unigene10040 | | 573 | | 2.77 |
| 124 | Unigene0030198 | 1983 | 6.15 | Unigene26405 | | 1902 | | 47.98 |
| 129 | Unigene0032251 | 857 | 6.45 | Unigene23582 | | 354 | | 3.80 |
| 131 | Unigene0035768 | 1330 | 5.61 | Unigene19393 | | 1658 | | 9.46 |
| 136 | Unigene0043584 | 1791 | 17.99 | Unigene10874 | | 835 | | 17.22 |
| 141 | Unigene0061887 | 333 | 0.97 | Unigene23256 | | 250 | | 10.77 |
| 142 | Unigene0063951 | 661 | 1.33 | Unigene24053 | | 1095 | | 7.56 |
| 23 | Unigene0027693 | 1745 | 2.60 | CL3301.Contig1 | | 1313 | | 8.87 |
|  | Unigene0041201 | 1156 | 59.72 | CL3301.Contig2 | | 1926 | | 117.40 |
|  | Unigene0042043 | 1831 | 102.96 | CL3301.Contig3 | | 1879 | | 66.50 |
| 36 | Unigene0021165 | 384 | 2.36 | Unigene29239 | | 246 | | 3.91 |
|  | Unigene0023705 | 482 | 0.91 | Unigene4483 | | 364 | | 16.25 |
|  | Unigene0027134 | 598 | 13.40 |  | |  | |  |
| 47 | Unigene0064401 | 212 | 0.69 | CL1221.Contig1 | | 269 | | 2.15 |
|  |  |  |  | CL1221.Contig2 | | 1808 | | 0.37 |
|  |  |  |  | CL1221.Contig3 | | 1799 | | 0.75 |
| 48 | Unigene0028538 | 260 | 53.31 | CL163.Contig1 | | 219 | | 7.69 |
|  | Unigene0053205 | 1604 | 2.41 | Unigene20949 | | 1668 | | 73.30 |
| 71 | Unigene0040531 | 1820 | 11.41 | CL3738.Contig3 | | 2899 | | 121.58 |
|  | Unigene0040532 | 1775 | 21.76 |  | |  | |  |
| OrthMCL | *I. pubescens* | | | *I. asprella* | | | | |
|  | Name | Length | RPKM | Name | Length | | RPKM | |
| 73 | Unigene0039415 | 1169 | 38.57 | CL955.Contig1 | 1657 | | 6.36 | |
|  | Unigene0039417 | 1961 | 54.53 |  |  | |  | |
| 74 | Unigene0049300 | 1904 | 30.49 | CL972.Contig1 | 1861 | | 37.40 | |
|  | Unigene0049301 | 1396 | 8.73 |  |  | |  | |
| 78 | Unigene0011775 | 589 | 1.14 | Unigene17547 | 259 | | 2.60 | |
|  | Unigene0055464 | 653 | 1.21 |  |  | |  | |
| 82 | Unigene0022897 | 1399 | 3.22 | CL1330.Contig1 | 1033 | | 5.59 | |
|  | Unigene0043249 | 1720 | 3.59 |  |  | |  | |
|  | Unigene0053162 | 1972 | 139.80 |  |  | |  | |
|  | Unigene0034040 | 1808 | 7.57 |  |  | |  | |
